# Transcriptional mechanism by which IS*5* activates the *fucAO* operon in *Escherichia coli*

**DOI:** 10.1093/nar/gkaf172

**Published:** 2025-03-11

**Authors:** Harry Zhou, Zhongge Zhang, Juan Velo, Jialu Huo, Sofia Smith, Allyson Ho, Milton H Saier

**Affiliations:** Department of Molecular Biology, University of California at San Diego, La Jolla, CA 92093, United States; Department of Molecular Biology, University of California at San Diego, La Jolla, CA 92093, United States; Department of Molecular Biology, University of California at San Diego, La Jolla, CA 92093, United States; Department of Molecular Biology, University of California at San Diego, La Jolla, CA 92093, United States; Department of Molecular Biology, University of California at San Diego, La Jolla, CA 92093, United States; Department of Molecular Biology, University of California at San Diego, La Jolla, CA 92093, United States; Department of Molecular Biology, University of California at San Diego, La Jolla, CA 92093, United States

## Abstract

The silent *E. coli fucAO* operon can be activated by IS*5* insertion upstream of its regulatory region, allowing cellular growth on L-1,2-propanediol. Little information is available concerning the transcriptional mechanism behind IS*5*-mediated *fucAO* activation. In this study, we demonstrate the formation of a unique “fusion” promoter (P*_fsn_*) following IS*5* insertion, which drives expression of the downstream *fucAO* operon. Our findings indicate that this functional σ^70^ fusion promoter is generated using a DNA sequence carrying a Crp-binding site directly upstream of the IS*5* element, followed by the otherwise inactive IS*5* transposase promoter. Under non-inducing conditions, this fusion promoter contributes to full operon expression while the native operon promoter P*_fucAO_* remains silent. As a typical Class I promoter, P*_fsn_* is independent of the *fuc* regulon activator FucR, but its activity is exclusively reliant on the binding of Crp-cAMP to the upstream Crp-binding site. Under inducing conditions, the presence of functional FucR can further elevate *fucAO* operon expression by activating the native operon promoter, P*_fucAO_*. In the latter case, P*_fsn_* and P*_fucAO_* function independently, and contribute to operon expression to nearly the same extent. Thus, we have discovered a novel IS-dependent fusion expression system that is modulated by a transcriptional factor in bacteria.

## Graphical Abstract

**Graphical Abstract:**
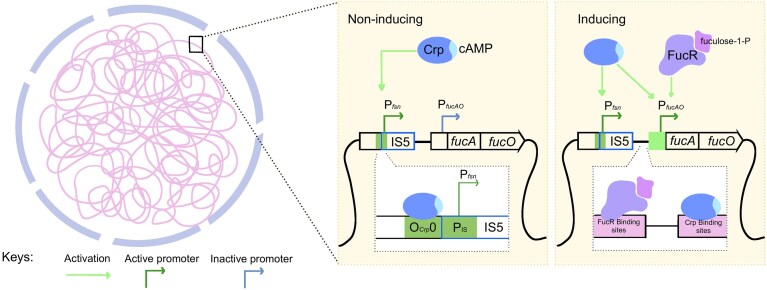

## Introduction

**Figure 1.**
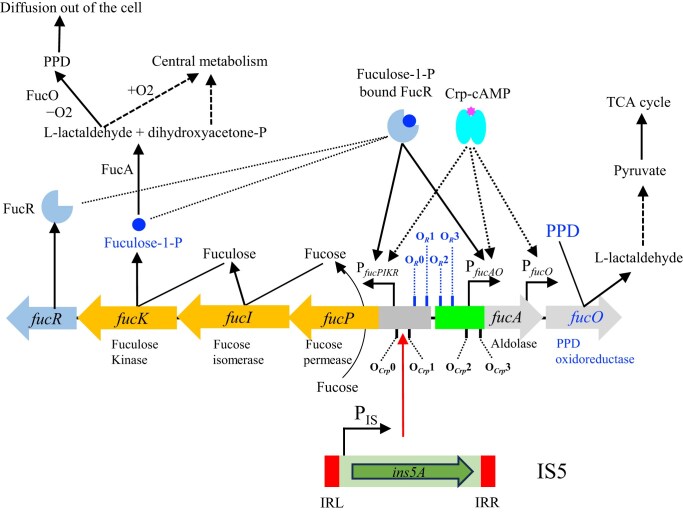
Organization of the *fuc* regulon and its regulation. The *fuc* regulon consists of the *fucPIKR* operon and the *fucAO* operon. The *fucPIKR* operon is driven by the operon promoter P*_fucPIKR_*, which is activated by Crp-cAMP bound to Crp operators O*_Crp_*0 and O*_Crp_*1, and FucR bound to putative FucR operators O*_R_*0 and O*_R_*1. The *fucAO* operon is driven by the operon promoter P*_fucAO_*, which is activated by Crp-cAMP bound to Crp operators O*_Crp_*2 and O*_Crp_*3, and FucR bound to FucR operators O*_R_*2 and O*_R_*3 [4, 7, 47, 57]. FucR becomes active and is capable of binding to its operators once complexed with the *fuc* regulon inducer, fuculose-1-P, an intermediate of the L-fucose pathway. In addition to P*_fucAO_*, the *fucO* gene is also driven by a weak promoter P*_fucO_* within the *fucA* gene [3], which is activated by Crp-cAMP but is independent of FucR [4]. The L-fucose pathway is mediated by the L-fucose permease (FucP), L-fucose isomerase (FucI), L-fuculose kinase (FucK) and L-fucolose-1-phosphate aldolase (FucA). In the presence of L-1,2-propandiol (PPD), the oxidoreductase (FucO) oxidizes PPD to L-lactaldehyde, which is converted to L-lactate and then pyruvate before entry into the TCA cycle. However, *E. coli* cells are PPD^−^ (unable to grow on PPD) because the *fucAO* operon is silent in the absence of L-fucose. Upon IS5 insertion upstream of P*_fucAO_* between O*_Crp_*0 and O*_Crp_*1, the *fucPIKR* operon is inactivated but the *fucAO* operon is activated, leading to high-level *fucO* expression that enables a PPD^+^ phenotype..

*Escherichia coli* can use L-fucose (6-deoxy-L-galactose, a methyl pentose) as a sole carbon and energy source for growth, both aerobically and anaerobically. As shown in Fig. 1, the L-fucose pathway is initiated by the L-fucose permease (FucP), followed by L-fucose isomerase (FucI), L-fuculose kinase (FucK), and L-fuculose-1-phosphate aldolase (FucA), encoded by two divergently oriented operons within the fucose regulon: *fucPIK* and *fucAO* [1, 2]. After uptake of fucose, the isomerase FucI converts L-fucose to L-fuculose that is phosphorylated by the kinase FucK, generating L-fuculose-1-phosphate. Subsequently, the aldolase FucA cleaves this methyl pentose derivative into two three-carbon intermediate metabolites: L-lactaldehyde and dihydroxyacetone-phosphate. Dihydroxyacetone-phosphate is an intermediate of glycolysis, which therefore enters central metabolism under both aerobic and anaerobic conditions. L-lactaldehyde represents a branching point in the L-fucose metabolic pathway. Under aerobic conditions, L-lactaldehyde is first oxidized to L-lactate by lactaldehyde dehydrogenase, which is further oxidized to pyruvate before entering general metabolism. Under anaerobic conditions, L-lactaldehyde is reduced to L-1,2-propanediol (PPD) by the *fucO*-encoded lactaldehyde:propanediol oxidoreductase (hereafter referred to as propanediol oxidoreductase) with NADH as a cofactor. PPD is excreted into the medium as a byproduct. Conceivably, this fermentation process does not generate energy for bacterial growth. Therefore, to grow on L-fucose anaerobically, only one triose intermediate, dihydroxyacetone-phosphate, enters central metabolism.

Among all the proteins within the *fuc* regulon, only FucO is needed for *E. coli* growth on propanediol (Fig. 1). Transcription of *fucO* is driven primarily by the *fucAO* operon promoter P*_fucAO_*. In addition, Li *et al.* reported the presence of a promoter (P*_fucO_*) within the 3′ end of *fucA*, driving *fucO* transcription [3]. However, this latter promoter was found to be weak and non-inducible [4]. The oxidoreductase FucO is capable not only of reducing L-lactaldehyde to PPD but also of oxidizing PPD back to L-lactaldehyde. However, *E. coli* cells cannot use PPD aerobically as a sole carbon source for growth due to the following reasons. First, the *fucAO* operon is embedded within a transcriptionally repressed locus on the chromosome [4], leading to overall low-level expression under various conditions. Second, *fucAO* operon expression cannot be activated when PPD serves as the sole carbon source since the *fucR* gene, encoding the *fuc* regulon activator, FucR, is not expressed, and the ligand fuculose-1-P (an intermediate metabolite of the fucose pathway), required for FucR activation, is absent. Third, propanediol oxidoreductase (FucO), an iron-dependent metalloenzyme that is residually produced in low amounts, becomes oxidatively inactivated in the presence of oxygen [5, 6]. Therefore, using wild-type cells, high-level *fucO* expression can only be achieved in the presence of fucose, under which conditions high levels of self-activated FucR and fuculose-1-P are available.

Wild-type *E. coli* cells can mutate to utilize PPD for aerobic growth (PPD^+^) after prolonged incubation with PPD [7, 8]. PPD^+^ mutants, which can readily arise on minimal PPD agar plates, carry an insertion sequence (IS) element, IS*5*, inserted into the *fucPIK*/*fucAO* intergenic region. As a transposable element, IS5 preferentially inserts into the target site with consensus sequence CA/TAG/A, which is subsequently duplicated on the other end of the inserted element [9, 10]. This is the case for all the PPD^+^ mutants, in which the inserted IS*5* is always flanked by its target site CTAG (−374 to −371 with respect to the *fucA* translational start site) located between Crp-binding sites O*_Crp_*0 and O*_Crp_*1 (Fig. 1). Furthermore, the IS5 element is always oriented in the same direction as the downstream *fucAO* operon in each identified PPD^+^ mutant [11]. IS*5* insertion leads to high-level expression of the *fucAO* operon, thereby synthesizing propanediol oxidoreductase at a level that renders the cells capable of aerobic growth on PPD [7, 12]. IS*5*-mediated *fucAO* expression is thought to be constitutive, being independent of two major activators FucR and Crp [7], the only known regulators for the *fuc* regulon. Meanwhile, IS*5* insertion abolishes expression of the *fucPIK* operon due to the displacement of the second Crp-binding site O*_Crp_*1 [13], leading to a negative phenotype on fucose. Using this unique *fuc* system, in which IS*5* insertion and excision lead to two distinct phenotypes, IS*5* has been shown to be able to excise from its insertion site [11].

Several studies have demonstrated that IS*5* transposition usually occurs at greater frequencies in response to stressful environmental conditions, and such a transpositional event is DNA structure-dependent [14] and is regulated by DNA binding proteins [15, 16]. The first well documented example of IS*5* insertional activation was IS*5* insertional mutation upstream the *glpFK* operon, which solely occurred in the presence of glycerol but was blocked by GlpR and Crp, two major regulators of *glpFK* operon expression that bind to a downstream region near the IS*5* insertion site [17, 18]. IS*5* transposition into the regulatory region of the cryptic *bglGFB* operon, leading to a β-glucoside growth positive phenotype, is induced by the presence of a β-glucoside. This insertional process is mediated by the operon anti-terminator BglG, probably via changing the DNA conformation, and thus facilitating IS*5* insertion [19]. Another example involves IS*5* insertion into the *flhDC* regulatory region, which occurs preferentially in *E. coli* cells growing within soft agar but not on hard agar or in liquid media [20, 21].

There are various ways by which the IS*5* element activates or elevates expression of neighboring target genes, including (i) de-repression by impairing the binding of specific transcriptional repressors [22–25]; (ii) native promoter activation by modifying the local DNA conformation and thus helping to recruit RNA polymerase [17, 26]; and (iii) disruption of H-NS-mediated DNA looping [27]. However, little has been known as to how IS*5* insertion activates the *fucAO* operon and why IS*5* is always inserted at the same site and in the same orientation in all identified IS*5*-activated PPD^+^ mutants. In addition, it is unknown whether and how FucR and cAMP-Crp (two positive regulators of the *fuc* regulon) and the native operon promoter P*_fucAO_* are involved in IS*5* activation of the operon.

Here we show that IS*5* insertion activates the *fucAO* operon by forming a functional “fusion” promoter, consisting of a short genomic region with a Crp-binding site upstream of the IS*5* insertion site and the silent IS*5* transposase promoter. Under non-inducing conditions, this fusion promoter contributes to expression of virtually the entire *fucAO* operon while the native operon promoter remains inactive. As a σ70 promoter, it is a typical Class I promoter as its activity depends on Crp binding to an upstream site, O*_Crp_*0, centered at position −61.5. The fusion promoter is FucR-independent. Under inducing conditions, functional FucR can further enhance *fucAO* operon expression by activating the native operon promoter.

## Materials and methods

### *E. coli* strains and growth conditions

*E. coli* K12 strains BW25113 [28] and ZZ200 (BW25113 deleted for *lacI*, *lacZ*, and *lacY*) [4] were used as wild-type strains. All other strains used in this study were derived from these strains, and they are described in Supplementary Table S1.

Bacterial strains were routinely grown in LB media at 30°C or 37°C. To test a propanediol growth positive (PPD^+^) phenotype, minimal M9 agar plates with 1% propanediol as the sole carbon source were used. To measure *fucAO* operon activities, test strains were cultured in M63 minimal media [29] with 0.5% (w/v) glycerol, 0.5% (w/v) L-fucose, or 0.5% (w/v) glucose as the carbon source. The 10× M63 salts solution contained 15 mM of (NH4)2SO4, 100 mM of KH2PO4, and 2 × 10–2 mM of FeSO4·7H2O. After diluting to 1x M63 medium, it was supplemented with 10–4% thiamine and 1.7 mM MgSO4. When necessary, ampicillin (Ap), kanamycin (Km), and chloramphenicol (Cm) were added to the media to 100, 25, and 10 μg/ml, respectively.

### Construction of *fucAO* operon transcriptional *lacZ* reporter strain ZZ224

In the *fucAO* operon reporter strain ZZ204 derived from ZZ200 [4], *fucA*, *fucO*, and *lacZ* (plus its own ribosome binding site, RBS) form a new operon driven by the *fucAO* promoter P*_fucAO_*. Following *lacZ*, there is a *cat* gene (encoding Cm resistance) driven by its own promoter. In strain ZZ204, the “*lacZ*:*cat*” cassette is located downstream of *fucO*, substituting for a 15-bp region (tgatgtgataatgcc) between the 5th and the 22nd nucleotides relative to the *fucO* stop codon. Using M9 + PPD minimal agar plates with Cm, this operon reporter cassette (*fucA*-*fucO*-*lacZ*::*cat*) was P1 transduced to strain PPD^+^Δ*lacZ*, yielding strain ZZ224. In this resultant reporter strain, IS*5* insertion is expected to activate the newly formed operon consisting of *fucA*, *fucO*, and *lacZ*, and thus, the transcription of *lacZ* truly represents the rate of *fucAO* operon transcription.

### Deletion of various parts of IS*5* and various parts of the upstream genomic region from ZZ224 and insertion of *km^r^* upstream P*_fucAO_* in ZZ204

To determine if operon activation is IS*5* sequence dependent (or is just activated by any DNA fragment insertion), using the Lambda-Red protocol [28], the entire IS*5* plus the upstream CTAG was replaced by a *km^r^* gene that is flanked by FRT (FLP recombinase recognition target) sites. The *km^r^* gene was subsequently eliminated (flipped out) using the FLP recombinase encoded by the helper plasmid pCP20, yielding strain ZZ225, in which the IS*5* element is replaced by an 85-bp FRT sequence (FRT scar). To further see if the IS*5* effect on *fucAO* is sequence specific, a *km^r^* gene was inserted at the same site in strain ZZ204 as for IS*5* in ZZ224, yielding strain ZZ226 (carrying a *km^r^* gene instead of IS*5* at the same chromosomal location).

The 177-bp IB (internal bent DNA) region, located in the 3′ end of IS*5*, consists of multiple permanent A-tracts and an IHF binding site in the center. To examine if this region alone activates the *fucAO* operon, the first 918 bp of IS*5* plus the upstream CTAG was replaced by a *km^r^* gene in the opposite direction, yielding strain ZZ227. The *km^r^* gene was flipped out, yielding strain ZZ228. Both ZZ227 (Km resistant) and ZZ228 (Km sensitive) each harbors the IB region only.

The left-side end of IS*5* carries a promoter-like region (P_IS_) that drives the transposase gene *ins5A* [30]; this promoter has almost no detectable activity [16]. To test if this promoter has any effect on IS*5* activation of *fucAO*, the first 68 bp region, located on the left end of IS*5* in strain ZZ224, was replaced by a *km^r^* gene that was subsequently removed, yielding strain ZZ229.

To determine if the chromosomal region upstream of CTAG, two short upstream regions, Up1 and Up2, located at −107 to −1 and −208 to −108, respectively, relative to the beginning of IS*5*, were individually replaced by a reversely oriented *km^r^* gene that was subsequently flipped out in strain ZZ224, yielding strains ZZ230 and ZZ231. These strains still have the intact IS*5* but lack a 100-bp upstream genomic region. To determine if the upstream genomic region (Up1 and Up2) itself has promoter activity, a *lacZ* gene plus its own RBS was substituted for IS*5*, P*_fucAO_* and *fucAO* in strain ZZ224, yielding strain ZZ232, in which *lacZ* is solely driven by the genomic region upstream of IS*5*.

### Construction of reporter strains for direct measurement of the hybrid promoter activity

Upon insertion, IS*5* and the upstream genomic region might have formed a new hybrid promoter (P*_fsn_*). To test such a possibility, the same “*lacZ*:*cat*” cassette (note *lacZ* has its own RBS) as for ZZ224 was integrated immediately upstream of the *ins5A*’s first codon in strain PPD^+^Δ*lacZ*, while the rest of IS*5*, P*_fucAO_* and the *fucAO* operon were deleted. This yielded strain ZZ233, in which *lacZ* is solely driven by the possible hybrid promoter P*_fsn_*.

To see if other portions of IS*5* had an additional effect on P*_fsn_* activity, the same “*lacZ*:*cat*” cassette was moved immediately downstream of the second CTAG site, replacing P*_fucAO_* and *fucAO*. This yielded strain ZZ234, in which *lacZ* is driven by P*_fsn_* together with the rest of IS*5* (promoter-less IS*5*). In addition to the use for direct measurement of P*_fsn_* activity, strains ZZ233 and ZZ234 can also be used to determine if the native promoter P*_fucAO_* plays a role in operon transcription.

### Insertion of an *rrnB* terminator downstream of P*_fsn_* and between P*_fucAO_* and *fucA*

In PPD^+^ strains such as ZZ224, there are two promoters (P*_fsn_* and P*_fucAO_*) driving *fucAO* expression. To further dissect the roles of these promoters, a *rrnB* terminator together with a *km^r^* marker was amplified from pKDT [31] and was then inserted immediately downstream of P_IS_ in strain ZZ224. The *km^r^* gene was flipped out, yielding strain ZZ235, in which P*_fsn_* was blocked by the inserted terminator. To block both P*_fsn_* and P*_fucAO_*, an *rrnB* terminator was added between P*_fucAO_* and the *fucA* start codon, yielding strain ZZ236.

### Mutation of O*_Crp_*0 upstream of *P_fsn_*

To determine the effect of Crp on P*_fsn_*, the operon reporter together with the upstream IS*5* was P1 transduced to a Glp^+^*crp* deletion strain [17], yielding strain ZZ237 that is the same as ZZ224 except that the *crp* gene is deleted.

There is one Crp-binding site (O*_Crp_*0) located upstream of the inserted IS*5* element in ZZ224, with nucleotide sequence “taa**TaTGA**cggcgg**TCACA**ctt” (two binding motifs are in bold while the nucleotides identical to the consensus are capitalized). This is one of two Crp-binding sites important for *fucPIKUR* operon transcription. To see if this Crp-binding site is required for P*_fsn_* activity in strain ZZ224, its sequence was changed to taa**Tacct**cggcgg**atgtt**ctt (the altered nucleotides are underlined and bolded) using fusion PCR followed by using the Lambda-Red approach. Prior to fusion PCR, a *km^r^* gene together with an *rrnB* terminator (*km^r^*:*rrnB*T) was amplified from pKDT_Pu [31] using primers Up1-km-F and Km-T-R (Supplementary Table S2). The Up1 fragment with the desired O*_Crp_*0 mutations (Up1-O*_Crp_*0) was amplified from BW25113 genomic DNA using primers Ocrp-mut-F and Ocrp-mut-R. These two fragments were fused together by PCR using Up1-km-F and Ocrp-mut-R, and the fusion products were chromosomally integrated in strain ZZ230. The resultant strain ZZ238 is the same as ZZ224 except that O*_Crp_*0 was altered. Similarly, the same mutation was made in O*_Crp_*0 upstream of P*_fsn_* in strain ZZ233, yielding strain ZZ239, in which P*_fsn_* alone drives *lacZ*.

### Construction of P*_fsn_* driving *lacZ* at the *lac* locus

To examine if and how FucR affects P*_fsn_* activity, one prerequisite is the presence of a functional FucR protein in the cell cytoplasm. Strains ZZ224 (complete IS*5*) and ZZ233 (P*_fsn_* alone) cannot make a functional FucR as they are Fuc^−^. To make this regulatory protein available and functional, it is necessary to make a new reporter strain, in which P*_fsn_* drives *lacZ* at a different locus while the native *fuc* regulon is untouched. To do so, a fragment carrying P*_fsn_*, the *lacZ*’s RBS and the first 200-bp region of *lacZ* (P*_fsn_*-*lacZ*’) was amplified from strain ZZ233 and then fused to the 3′ end of the “*km^r^*:*rrnB*T” fragment (see above). The fusion product was integrated onto the chromosome of BW25113 to replace the *lacI* gene and the *lacI*/*lacZ* intergenic region. This yielded strain ZZ240, in which P*_fsn_* alone drives *lacZ* at the *lac* locus while the native *fuc* regulon is intact. When this strain is cultured with fucose, the cells are expected to synthesize functional (activated) FucR like wild-type strain BW23113.

To further confirm the dependence of P*_fsn_* on Crp, the sequences of the −35/−10 motifs in P*_fsn_* were changed to the consensus sequences (TTGACA for the −35 motif and TATAAT for the −10 motif). To make such changes, the P*_fsn_* region carrying Up1 and P_IS_ in strain ZZ233 was first replaced by a *km^r^* gene that was then flipped out, leaving an 85-FRT scar. Using the cells of ZZ239 (P*_fsn_* with ΔO*_Crp_*0) and pKDT as the templates, two fragments were amplified, one carrying the *km^r^* gene, *rrnB*T and the first part of P*_fsn_* and the other carrying the second part of P*_fsn_*, the *lacZ*’s RBS and the first 120 bp of *lacZ*. These two fragments shared a 42-bp region that harbors the modified −35/−10 elements matching the consensus sequences. These fragments were fused together by fusion PCR and subsequently integrated into the chromosome to replace the 85-bp scar in strain ZZ233 deleted for Up1 and P_IS_. This yielded strain ZZ242 in which the modified P_IS_ with consensus −35/−10 motifs (referred to as P*_cons_*), together with a mutated upstream O*_Crp_*0, drives *lacZ* at the *fuc* locus.

To see if the upstream Crp-binding site O*_Crp_*0 had an effect on P*_cons_*, the native O*_Crp_*0 site was added back upstream of P*_cons_* in ZZ242. To do so, two fragments with the desired substitutions were amplified from strain ZZ233 (P*_fsn_* plus the native O*_Crp_*0) and pKDT. These two fragments were combined using fusion PCR and subsequently integrated into the chromosome of strain ZZ233 deleted for Up1 and P_IS_), yielding strain ZZ243, in which P*_cons_* (with the consensus −35/−10 motifs), together with the upstream O*_Crp_*0, drives *lacZ* at the *fuc* locus.

### Screening PPD^+^/Fuc^+^ double mutants

IS*5* insertional PPD^+^ mutants such as ZZ224 are Fuc^−^. To obtain PPD^+^/Fuc^+^ double mutants, PPD^+^ cells (about 10^8^ cells per plate) were applied onto minimal M9 agar plates with fucose as the sole carbon source. The plates were incubated at 30°C, and some colonies (PPD^+^/Fuc^+^ double mutants) appeared after a 5-day incubation. Several mutants were purified, and the *fucPIK*/*fucAO* regions plus the IS*5* element were subject to sequencing analyses. One such mutant, named ZZ241, carried an inserted single nucleotide “T” immediately downstream of O*_Crp_*0 (that is, −146 with respect to the *fucP*’s start codon). No other mutations were found within the IS*5* element and the entire *fuc* regulon. Strain ZZ241 was used to determine if the functional FucR protein has an additional impact on *fucAO* expression in IS*5* insertional PPD^+^ cells.

### β-galactosidase (LacZ) activity assay

A β-galactosidase (LacZ) assay can be separated into two main parts: sample preparation and the enzymatic assay. For sample preparation, a fresh colony of the reporter strain was cultured in 5 ml of LB medium at 37°C with shaking for about 6 hours. The culture (30 μl) was transferred to another tube containing 3 ml of minimal M63 media with 0.5% glycerol or 0.5% fucose as the sole carbon source. The M63 culture (preculture) was then left to grow overnight at 37°C with shaking. The next day, a specific amount of overnight culture (preculture) was inoculated into 5 mL of the same M63 media to OD600 of 0.03, and the culture was grown at 37°C with shaking. During the exponential growth phase, at least four samples were collected in an OD600 range of 0.2 to 1.0. Collected samples were immediately frozen at −20°C prior to the assay.

For the assay, the previously frozen samples were first thawed at room temperature. Around 200 μl of sample, 800 μl of LacZ-buffer, and 25 μl of chloroform were combined in a small glass tube and vortexed twice for 10 s each. The sample tubes were placed into a water bath incubator and warmed to 37°C. To initiate the reaction, 200 μl of o-nitrophenyl-β-D- galactopyranoside (β-ONPG) was added to each sample. After the yellow color had visibly developed, 0.5 ml of 1M sodium carbonate was added to each sample and vortexed to stop the reaction. The reaction mixture was appropriately diluted and then centrifuged for 2.5 min at 15 000 rpm. Absorbance values of the prepared reaction mixture were measured at 420 and 550 nm. The β-galactosidase (LacZ) activity for each sample was then calculated using the formula: β-galactosidase (LacZ) activity (Miller units) = [1000 x (OD_420nm_ – 1.75 x OD_550nm_) x Dilution factor] / [Time of reaction (min) x Volume of sample (ml)]. The slope of LacZ activities versus the collected OD600 values for each sample represents the reporter strain activity. The final activity was the average of at least three repeats (that is, at least 12 samples per strain).

### TSS determination using a SMARTer® RACE 5′/3′ Kit

To prepare total RNA, the strain of interest was shaken at 37°C in M63 minimal media with 0.5% glycerol as the carbon source. When the OD_600_ reached about 1.0, a 600 μl culture was mixed (by vortexing) with 1.2 ml RNAprotectTM Bacteria Reagent (Qiagen) in a 2.0 ml microcentrifuge tube. After 5 min of incubation at RT, the mixture was centrifuged at 5000 rpm for 10 min, and the pellet was air dried for 5 min before being frozen at −20°C. A NucleoSpin® RNA Kit (Takara Bio USA, inc.) was used to extract the total RNA from the frozen cell pellet. The pellet was first lysed by lysozyme (1mg/ml) and subsequently bound to the NucleoSpin Filter. The NucleoSpin filter was desalted, treated by the provided rDNase (to remove residual DNA), washed and dried prior to RNA elution with RNase-free deionized water. The eluted total RNA sample was stored at −80°C, and its absorbance ratios 260/280 and 260/230 of the eluted RNA sample were measured using a NanoDrop (Thermo Scientific NanoDrop 1000) to ensure RNA purity.

mRNA was extracted using a MICROB*Express*TM Bacterial mRNA Purification Kit (Invitrogen). The total RNA sample was thawed slowly on ice, mixed with 100% ethanol, and centrifuged (12 000 rpm for 30 min) at 4°C. The resulting RNA pellet was washed (three times using 70% ethanol), air dried and dissolved in >15 μl TE buffer (containing 10 mM Tris-HCl and 1 mM EDTA at pH 8.0). RNA was then introduced to the provided binding buffer with Capture Oligo Mix. The mixture was incubated at 70°C for 10 min and 37°C for 30 min to denature the 16S and 23S rRNAs and facilitate hybridization of the rRNAs to capture oligonucleotides. The mixture was combined with the MagBeads and was incubated for 15 min at 37°C to allow the MagBeads to anneal to the hybridized oligonucleotides bound to the rRNA. The MagBead slurry was then placed in a magnetic stand to draw the MagBeads from the solution, leaving the supernatant. The mRNA present in the supernatant was precipitated using 5 mg/mL glycogen, 3M sodium acetate, and 100% ethanol. After centrifugation (13K rpm, 30 min), the mRNA pellet was washed (with 70% ethanol), air dried briefly and dissolved in nuclease-free deionized water before storing it at −80°C.

5′ RACE was performed using the SMARTer® RACE 5′/3′ kit (Takara Bio USA). To synthesize first-strand cDNA, the extracted mRNA sample was combined with a random hexamer mixture that binds to the mRNA. The mixture was incubated at 72°C for 3 min and then at 42°C for 2 min. A buffer containing RNase inhibitor, Reverse Transcriptase, and SMARTer II Oligonucleotide (all provided) was added to the mixture which was subsequently incubated at 42°C for 90 min and then 70°C for 10 min. The resulting mixture (first-strand cDNA) was diluted with tricine-EDTA buffer. After dilution, the first-strand cDNA was combined with a PCR master mix, 5′ gene-specific primer, and the universal primer mix for amplification. PCR products (that is, amplified cDNA) were purified by gel electrophoresis, and the purified cDNA was subsequently sequenced. The first nucleotide immediately downstream of the SMARTer II Oligonucleotide sequence is the transcriptional start site (+1) of the target gene.

### Statistical analysis

All β-galactosidase activity data are expressed as the mean ± standard deviation (SD). The data points (represented by orange dots) used for mean and SD calculation are attached to bar graphs. Statistical significance was tested by either two-sample t-test (for two treatments) or 1-way ANOVA followed by Tukey Kramer's post hoc test (for ≥ 3 treatments). All figures and β-galactosidase activities were generated using Microsoft Excel (Version 16.66.1) or RStudio (Version 2023.12.0 + 369 “Ocean Storm” Release for Windows). Details of the statistical tests used are indicated in the figure legends. Sample size details are described in Section "β-galactosidase (LacZ) activity assay". ns denotes no significance and indicates a *P*-value ≥ 0.05; * indicates a *P*-value < 0.05; ** indicates a *P*-value < 0.01; *** indicates a *P*-value < 0.001; and **** indicates a *P*-value < 0.0001.

## Results

### Significance of IS*5* presence with respect to *fucAO* operon activity

**Figure 2.**
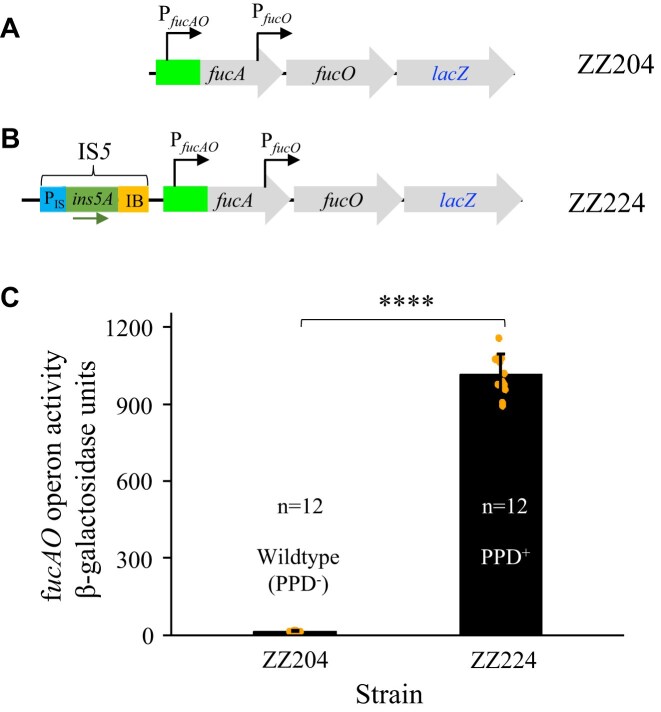
IS*5* insertion dramatically increases *fucAO* operon transcription. (**A**) Schematic diagram showing the *lacZ* transcriptional reporter for the *fucAO* operon in wild-type cells. A *lacZ* gene plus its RBS was integrated downstream of the *fucO* gene within the native *fucAO* operon while the native *lacZ* was deleted. P*_fucAO_* and P*_fucO_* denote the *fucAO* operon promoter and the *fucO* promoter, respectively. (**B**) Schematic diagram showing the *lacZ* transcriptional reporter for the *fucAO* operon in IS*5* insertional PPD^+^ mutant cells. IS*5* inserts at −374.5 upstream of the *fucA* translational start site. Within IS*5*, P_IS_, *ins5A* and IB denote the transposase promoter (the 68-bp 5′ end region of IS*5*), the transposase gene and the internal bend region located at the 3′ end of IS*5*, respectively. (**C**) The *fucAO* operon activities in wild-type and PPD^+^ cells. Test strains were cultured in M63 minimal media with shaking (200 rpm) at 37°C. At least four samples were collected at OD_600_ values of 0.2–1.0 during the exponential growth period. β-Galactosidase assays and enzyme activity measurements were as described in “Materials and methods.” The slope of OD_600_ values versus β-galactosidase activities was defined as the promoter activity. Data are plotted as the mean ± SD (two-sample t-test). The data points (represented by orange dots are attached to bar graphs. For all the figures, ns denotes no significance and indicates a *P*-value ≥ 0.05; * indicates a *P*-value < 0.05; ** indicates a *P*-value < 0.01; *** indicates a *P*-value < 0.001; and **** indicates a *P*-value < 0.0001.

In the absence of fucose, the *fucAO* operon is known to be inactive in wild-type cells prior to IS*5* insertion, upon which it is thought to be constitutively activated. The mechanism revealing how the insertional event causes operon activation is not fully understood. To investigate how IS*5* interacts with the *fucAO* operon, we first needed to choose a reliable and accurate method of measuring the operon activity by using LacZ as a reporter. Considering the presence of two promoters (P*_fucAO_* and P*_fucO_*), the latter driving only *fucO* expression, with a possible chromosomal location impact, we decided to introduce a *lacZ* gene (with its own RBS) immediately downstream of *fucO*, yielding a 3-cistronic operon consisted of *fucA*, *fucO* and *lacZ* (Fig. 2). Two such strains, ZZ204 [4] and ZZ224 (this study) were constructed using wild-type strain BW25113 (PPD^−^) and an IS*5* insertional mutant (PPD^+^) mutant respectively, where the native *lacZ* gene was removed (Fig. 2A and B). The operon activities were measured using standard β-galactosidase assays after growing these reporter strains in M63 media with 0.5% glycerol as the sole carbon source (hereafter referred to as the standard assay). Fig. 2C identifies a 75-fold increase in operon activity following IS*5* insertion between the two divergent operons, *fucPIK* and *fucAO*. In the wild-type strain, ZZ204, the measured operon activity was only about 14 LacZ units, a clear representation of the inactive nature of *fucAO*, eliciting a PPD^-^ phenotype. In the IS*5* insertional strain, ZZ224, the observed 1060 LacZ units corresponds to a PPD^+^ phenotype.

### The IS*5* sequence itself is necessary to illicit the PPD^+^ phenotype

**Figure 3.**
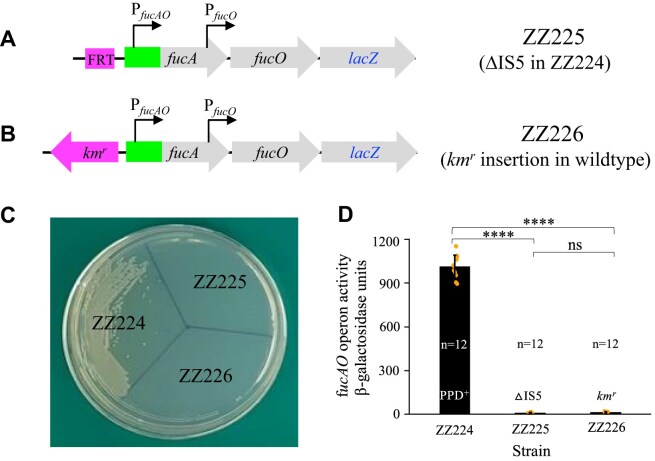
The IS*5* nucleotide sequence is important for *fucAO* operon activation. (**A**) Schematic diagram showing the deletion of IS*5* inserted upstream of P*_fucAO_* in reporter strain ZZ224. A kanamycin resistance gene (*km^r^*), which is flanked by the FLP recombinase recognition target (FRT) sites, was first substituted for IS*5* and then was eliminated (flipped out) leaving a remaining 85-bp FRT scar. (**B**) Diagram showing insertion of a *km^r^* gene in the opposite direction at −374.5-bp (the same site as IS*5* in ZZ224) upstream of the *fucA* translational start site in wild-type cells. (**C**) Growth assay using minimal M63 agar plates with PPD as the sole carbon source. Fresh colonies were cultured in LB with shaking at 37°C for 8 h. The cells were collected and washed twice using 1x M63 medium without a carbon source before being streaked onto the agar plates. The plates were incubated at 37°C for 2–3 days before being examined for colony sizes. (**D**) β-galactosidase assays showing *fucAO* operon transcription in non-inducing cells grown with glycerol as the carbon source. Data are plotted as the mean ± SD (one-way ANOVA with Tukey Kramer's post hoc test).

To determine if the nucleotide sequence of IS*5* is necessary for a PPD^+^ phenotype, a *km^r^* gene was used to first substitute for the inserted IS*5* element (and its upstream CTAG insertion site) in strain ZZ224 (carrying a *fucA*-*fucO*-*lacZ* operon in an IS*5* insertional PPD^+^ mutant), and then was subsequently eliminated by the helper plasmid pCP20, yielding strain ZZ225 (deleting IS*5* in ZZ224) (Fig. 3A). This procedure was performed in a wild-type background as well by inserting a *km^r^* gene immediately upstream of the IS*5* insertion site, CTAG, yielding strain ZZ226 (carrying a *km^r^* gene upstream of P*_fucAO_* at the same site as IS*5* in ZZ224) (Fig. 3B). As shown in Fig. 3C, deletion of the IS*5* element from a PPD^+^ mutant resulted in a PPD^-^ phenotype (see ZZ225). Insertion of a 1.3-kb *km^r^* gene (similar size to IS*5*) into the wild-type chromosome at the same site as for IS*5* did not alter its PPD^−^ phenotype (see ZZ226 in Fig. 3C).

The operon activities were measured in these strains cultured in glycerol minimal medium, and the results are summarized in Fig. 3D. As can be seen, strain ZZ224 (carrying the original IS*5*) exhibited normal operon activity (i.e. 1080 units of β-galactosidase activity), but the other two strains, ZZ225 (with IS*5* deletion) and ZZ226 (with *km^r^* insertion), only exhibited basal operon activities (about 15 units), consistent with their PPD^−^ phenotype. Sequencing of all these strains revealed that no additional mutations were present in the P*_fucAO_, fucAO*, or IS*5* regions that might have conferred a PPD^+^ phenotype. Based on these results, we conclude: (i) the IS*5* nucleotide sequence itself (not just any insertion) is necessary to activate the *fucAO* operon, thereby causing the PPD^+^ phenotype; (ii) IS*5* activation of *fucAO* is not due to de-repression since this operon is not repressed by any known repressors (instead, it is only regulated by two activators: FucR and Crp), and the insertion of a *km^r^* gene at the same site as IS*5* did not activate the operon; and (iii) IS*5* insertion might introduce a new promoter region, driving the downstream *fucAO* operon.

### The mechanism of IS*5* activation of *fucAO* is dissimilar from the IS*5* activation of *glpFK*

**Figure 4.**
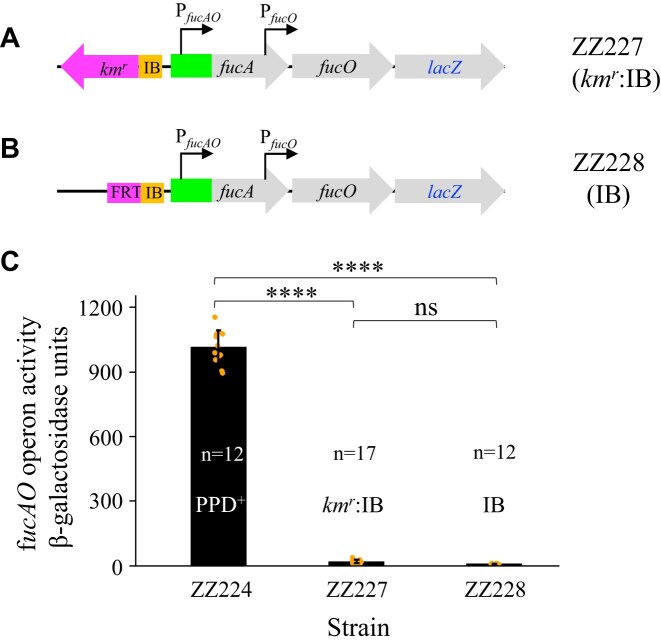
The internal DNA bend (IB) region of IS*5* has no impact on *fucAO* operon activation. (**A**) Schematic diagram showing the *km^r^* substitution for most of the inserted IS*5* element save for the IB region. IB refers to a 177-bp 3′ end region of IS*5* that carries A-tracts and a centrally localized IHF binding site. This region has the same capability as IS*5* to activate the *glpFK* operon [26]. A Km marker was first substituted for IS*5*, and it was then flipped out leaving an 85-bp FRT scar. (**B**) Diagram showing the presence of the 85-bp scar left after the *km^r^* gene was flipped out in strain ZZ227 shown in Fig. 3A. (**C**) β-galactosidase assays showing the IB effect on *fucAO* operon transcription under non-inducing conditions. Data are plotted as the mean ± SD (one-way ANOVA with Tukey Kramer’s post-hoc test).

As mentioned before, the *glpFK* operon is the most thoroughly studied example where IS*5* insertion affects the host operon activity. To activate either the *glpFK* operon or the *fucAO* operon, the IS*5* inserts must always occur at the same location upstream of the promoter region and in the single orientation (with the transposase gene transcribed in the same direction as the target operon). For the *glpFK* case, previous research showed that the 177-bp IB region at the 3′ end of IS*5* contributes to maximal *glpFK* expression [26]. In the two IB strains (ZZ227 and ZZ228) shown in Fig. 4A and B, we have removed most of the IS*5* element save for the IB region. The objective of constructing these two strains was to explore whether the IB region of IS*5* in *fuc* was comparable in operon expression levels to those found in *glpFK*. The resulting activity for both strains, with or without the Km marker, was observed to be equivalent to wild-type control levels (Fig. 4C), indicating that IS*5*-mediated activation of *fucAO* does not involve the IB region.

### The IS*5* transposase promoter and the genomic region upstream of IS*5* are important, but neither alone is sufficient, for full *fucAO* expression

**Figure 5.**
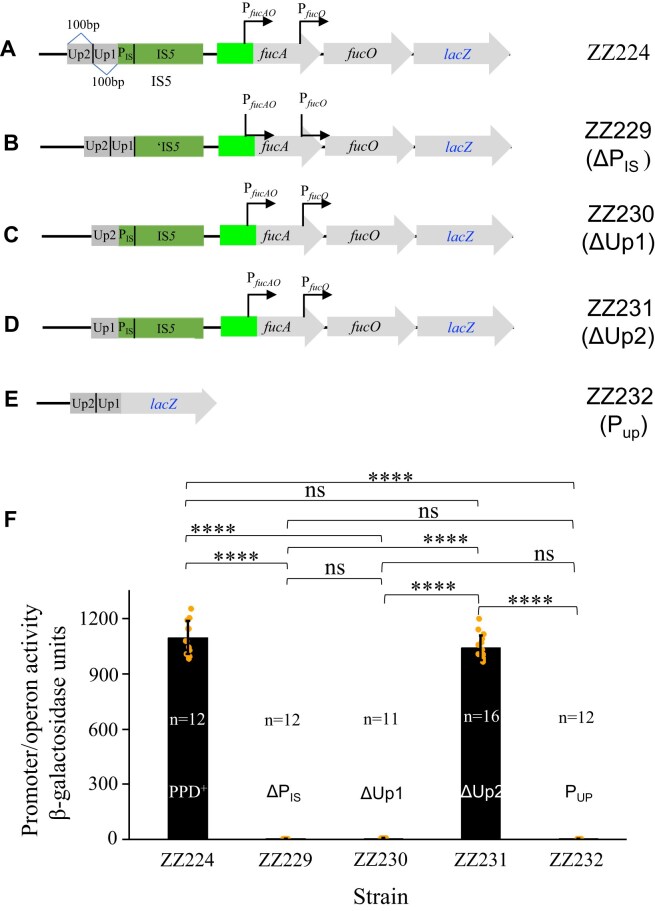
Effects of the IS*5* transposase promoter and upstream genomic regions on *fucAO* operon transcription. (**A**) Schematic diagram showing upstream genomic regions and the promoter (P_IS_) for the IS*5* transposase gene, *ins5A*. Up1 and Up2 denote two 100-bp genomic regions located upstream of IS*5*. (**B**) Diagram showing the deletion of P_IS_ in ZZ224 shown in (**A**). ‘IS*5* denotes a promoter-less IS*5* lacking the first 68-bp 5′ end region (P_IS_). (**C**) Diagram showing the deletion of Up1 in ZZ224 shown in (**A**). (**D**) Diagram showing the deletion of Up2 in ZZ224 shown in (**A**). (**E**) Diagram showing that upstream genomic regions directly drive *lacZ* expression. In this strain, ZZ232, the entire IS*5*, P*_fucAO_* and the *fucAO* operon are replaced by the *lacZ* gene with its own RBS. (**F**) β-galactosidase assays showing the effects of deleting P_IS_, Up1 or Up2 on *fucAO* operon transcription, and determining if the upstream genomic regions alone have promoter activity. Data are plotted as the mean ± SD (one-way ANOVA with Tukey Kramer's post-hoc test).

Since the downstream IB region is dispensable in *fucAO* operon activation, we directed our investigation toward the upstream regions including the IS*5* transposase promoter (P_IS_, referring to the 68-bp 5′ end region of IS*5*) and the genomic regions upstream of the IS*5* insertion (Up1 and Up2) (Fig. 5A). Strain ZZ229 was constructed where P_IS_ was removed from strain ZZ224 (Fig. 5B). Two other strains, ZZ230 and ZZ231, were constructed, where P_IS_ is intact but two 100-bp regions, −1 to −100 (Up1) and −101 to −200 (Up2) relative to the 5′ end of IS*5*, were deleted, respectively (Fig. 5C and D). To examine if the upstream genomic regions, including Up2 and Up1, have any promoter activity, strain ZZ232 was made by replacing the region carrying IS*5*, P*_fucAO_* and *fucAO* with a *lacZ* gene plus its own RBS (Fig. 5E).

Using the standard assay, the operon activities within these reporter strains were measured, and the results are summarized in Fig. 5F. Without P_IS_ (strain ZZ229), the operon expression was abolished, indicating that the promoter region of the IS*5* transposase gene is essential for *fucAO* expression, although it has little activity by itself. The same observation was made when the immediate upstream region (Up1) alone (leaving P_IS_ and Up2 intact) (strain ZZ230) was deleted, indicating that this Up1 region is indispensable for operon expression as well. However, deletion of a further upstream region (Up2) distal to IS*5* insertion (strain ZZ231) had a negligible effect on operon activation by IS*5*.

Since Up1 and P_IS_ are both essential for IS*5* activation of *fucAO* (see above), and P_IS_ alone has essentially no activity [16, 30], it is interesting to know if this Up1 site together with its upstream region has promoter activity. To do so, a new reporter strain ZZ232 was constructed in which the same *lacZ* reporter was directly driven by this genomic region (Fig. 5E). As shown in Fig. 5F, this upstream region (including Up2 and Up1) alone had no detectable promoter activity (the 1^st^ column on the right). Based on these results, several conclusions can be drawn: (i) both the transposase promoter (P_IS_) and its immediate upstream genomic region (Up1) are required for *fucAO* operon expression, since the absence of each of these regions abolishes high level operon expression; (ii) the further upstream region (Up2) is dispensable for IS*5* activation of the operon; (iii) neither P_IS_ nor Up1 alone is capable of operon expression, as the presence of each of these elements without the other has essentially no effect on *fucAO* expression; (iv) a joint effort between P_IS_ and Up1 is needed for full operon expression, suggesting the formation a functional fusion promoter. Thus, we defined a functional fusion promoter as an active two-part promoter including any necessary regulatory regions: one part from the adjacent genomic regions, and the other from the inserted IS element.

### Direct measurement of the proposed fusion promoter generated upon IS*5* insertion

**Figure 6.**
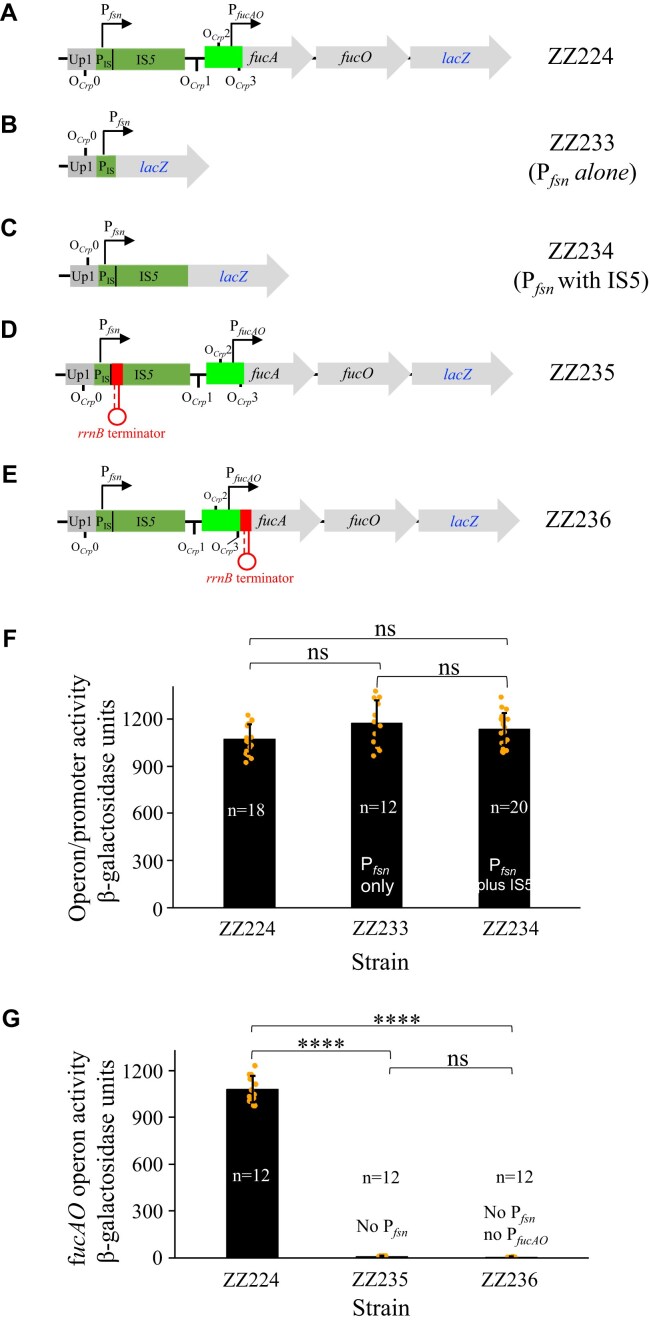
The fusion promoter alone is sufficient to activate the *fucAO* operon. (**A**) Schematic diagram showing the fusion promoter P*_fsn_*, the *fucAO* operon promoter P*_fucAO_* and four Crp-binding sites. (**B**) Diagram showing P*_fsn_* alone driving *lacZ* at the *fucAO* locus. (**C**) Diagram showing P*_fsn_* together with the rest of IS*5* driving *lacZ* at the *fucAO* locus. (**D**) Schematic diagram showing an inserted *rrnB* terminator (*rrnB*T) immediately downstream of P*_fsn_*. (**E**) Schematic diagram showing an inserted *rrnB* terminator (*rrnB*T) immediately downstream of P*_fucAO_*. (**F**) β-galactosidase assays, revealing that P*_fsn_* alone can sufficiently drive *fucAO* transcription under non-inducing conditions. (**G**) β-galactosidase assays revealing that blocking P*_fsn_* or both P*_fsn_* and P*_fucAO_* terminates *fucAO* transcription. Data are plotted as the mean ± SD (one-way ANOVA with Tukey Kramer’s post-hoc test).

As described above, the proposed fusion promoter (P*_fsn_*), formed upon IS*5* insertion, consists of an immediate upstream genomic region (Up1) and the beginning region (P_IS_) of IS*5* (Fig. 6A). To directly determine the activity of such a fusion promoter, the same *lacZ* cassette was added immediately downstream of P_IS_ on the chromosome of an IS*5* insertional PPD^+^ mutant, replacing the remaining part of IS*5*, P*_fucAO_* and *fucAO*. This yielded strain ZZ233 (Fig. 6B), in which the fusion promoter alone drives *lacZ* transcription at the *fuc* locus. To determine if the remaining part of IS*5* has an additional effect on P*_fsn_*, the *lacZ* cassette was integrated downstream of the inserted IS*5*, yielding strain ZZ234 (Fig. 6C), in which the entire IS*5* plus the same upstream genomic region drives *lacZ*.

The promoter activities were quantitated for strains ZZ233 and ZZ234 using our standard assay. As seen in Fig. 6F, the fusion promoter P*_fsn_* alone yielded 1225 units of β-galactosidase activity in strain ZZ233, about 15% higher than the entire *fucAO* operon activity observed for strain ZZ224 which harbors P*_fsn_* and the complete IS*5*, P*_fucAO_* and *fucAO*. This slightly greater activity observed for P*_fsn_* alone might be due to the presence of a downstream region that may partially block transcription (see Discussion). Meanwhile, no additional promoter activity was observed with the entire IS*5* present (that is, P*_fsn_* with the complete IS*5*) (strain ZZ234 in Fig. 6F).

To further confirm that P*_fsn_* is the only driver for *fucAO* operon expression, strains ZZ235 and ZZ236 (Fig. 6D and E) were made, which carry an *rrnB* terminator either downstream of P*_fsn_* (ZZ235) or downstream of P*_fucAO_* (ZZ236). When cultured with glycerol, strain ZZ235 expressed a basal level of *fucAO* (only 15 units of β-galactosidase activity) (Fig. 6G), like the wild-type strain without the IS*5* insertion, indicating that P*_fsn_*, when blocked by a terminator, failed to transcribe the *fucAO* operon. With strain ZZ236 (carrying a terminator downstream of P*_fucAO_*), a further decrease in operon expression (about 5 LacZ units) was detected due to the blockage of both P*_fsn_* and P*_fucAO_* by the terminator. Based on these results, we conclude: (i) the fusion promoter itself is active and contributes to essentially the entire *fucAO* operon expression within PPD^+^ cells; (ii) the remaining IS*5* region (over 90% of IS*5*) without P_IS_ does not exert a stimulatory effect on *fucAO* operon expression, and (iii) the downstream genomic regions including P*_fucAO_* and *fucAO*, appear to be slightly inhibitory to the operon expression in PPD^+^ cells.

### Identification of the transcriptional start site for P*_fsn_* generated upon IS*5* insertion

The experiments described above provided evidence for the formation of a fusion promoter (P*_fsn_*) upon IS*5* insertion, and this promoter clearly accounts for the high level of *fucAO* operon expression. To validate this finding, we performed 5′ RACE to identify the transcriptional start site + 1 (TSS) of P*_fsn_*, using reverse gene-specific primers (GSPs) to define the target region for amplification (see Methods).

We first used a GSP, GST-fucA-R (Supplementary Table S2), that binds to a downstream region near the *fucA* start codon. With this GSP, a single 1.65-kb amplified product was anticipated. However, no such band was observed (data not shown). One possible reason could be that the Takara kit was insufficient to amplify large DNA fragments, especially where DNA secondary structures are present (personal communication with Takara). Next, to make the proposed product smaller, we designed an IS*5*-specific GSP, GST-IS*5*-R (Supplementary Table S2), targeting a region downstream of the start codon of the IS*5* transposase gene, *ins5A*. With this GSP, a 0.3-kb DNA band was expected. However, we did not observe the proposed 0.3 kb band, and instead, some other unexpected bands were found (data not shown). These results indicate that the use of an IS*5*-specific GST may not be a good option as there are 11 copies of IS*5* in the genome of the *E. coli* strains [32] used in this study.

**Figure 7.**
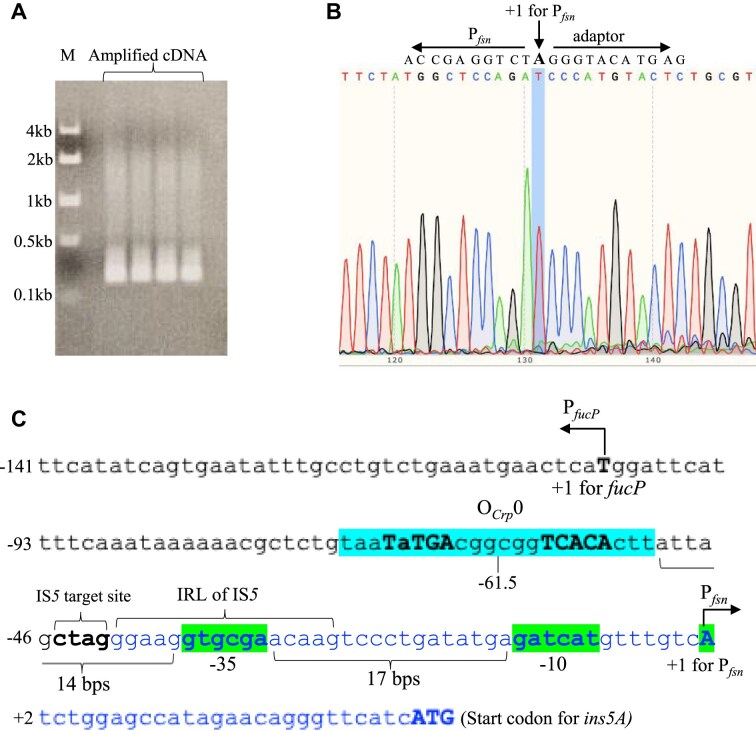
Determining the transcriptional start site (TSS) for P*_fsn_* using 5′ RACE. Strain ZZ233, harboring the P*_fsn_*-driven *lacZ* cassette at the *fuc* locus, was used for TSS determination. This strain was cultured under non-inducing conditions. Total RNA preparation, rRNA removal, mRNA purification, cDNA synthesis and amplification with a *lacZ* specific oligo (binding to the *lacZ* gene near its start codon) were described in Materials and Methods. (**A**) Agarose gel picture showing an about 0.2-kb cDNA product amplified using the *lacZ*-specific oligo. (**B**) Sequencing chromatograms of a part of the *lacZ* cDNA revealing the TSS. The capital “A” is the TSS determined for P*_fsn_* by 5′ RACE. The sequence on the left side of the TSS is the beginning sequence of the 5′ untranslated region of *ins5A* while the sequence on the right side is derived for a sequencing adaptor provided within the Takara Bio kit. (**C**) DNA sequence of P*_fsn_* with annotations. P*_fsn_* consists of two parts: upstream genomic region Up1 (-141 to -42) and *ins5A* promoter region (-41 to +30). Within the promoter region, the TSS (**A**), −10 element (gatcat) and −35 element (gtgcga) are green highlighted. The TSS and the −10 element are the same as ones proposed previously for the *ins5A* promoter P_IS_ [30]. The −35 element was proposed in this study, and it is 17 bp upstream of the −10 element. The IS*5* target site “ctaa” and the 16-bp IRL of IS*5* are marked. Embedded in the Up1 genomic region is a Crp-binding site, O*_Crp_*0, which is cyan highlighted with two binding motifs being bolded. O*_Crp_*0, centered at −61.5 relative to the TSS, is located 14 bps upstream of the −35 hexamer. The bold “T” is the TSS for *fucP* that is transcribed in the direction opposite to P*_fsn_*. The number on the left side of each row indicates the position of the first nucleotide with respect to the TSS.

The above observations suggest two criteria for determination of the TSS using the Takara kit: a small cDNA product (preferentially 0.2 to 0.3 kb in length), and the use of a non-IS*5* specific GST primer. To satisfy these preferences, strain ZZ233 (in which P*_fsn_* only drives *lacZ* at the *fucAO* locus (Fig. 6B and Supplementary Table S1) was used, with which the target region could be shortened to approximately 0.2 kb in length by using the GST primer GST-lacZ-R (Supplementary Table S2) that binds to a *lacZ* region near the start codon. This modification enabled the reverse transcriptase to successfully traverse to P*_fsn_*’s transcriptional start site (TSS), yielding a 0.2 kb DNA product as expected (Fig. 7A). The subsequent DNA sequencing analysis of this product by the same GST primer (GST-lacZ-R) revealed a specific ‘A’ nucleotide as the fusion promoter TSS (Fig. 7B), which was identical to the TSS of the *ins5A* promoter (Fig. 7C). This result is noteworthy since it indicates that the novel fusion promoter drives the transposase gene and the downstream *fucAO* operon using the same TSS.

The P*_fsn_* structure is shown on Fig. 7C. The −10 element and the TSS are the same as previously reported [30]. A −35-like element with sequence “gtgcga” was identified based on the fact that the optimal distances between the −10 motif and −35 motif are 17–18 bps for typical σ70 promoters. These three elements, −35, −10, and + 1, appear to form a core promoter driving *ins5A* expression although it is essentially silent by itself. Embedded in the upstream Up1 genomic region, a Crp-binding site O*_Crp_*0, centered at −61.5 with respect to the TSS, is present upstream of the −35 hexamer. Therefore, the fusion promoter, formed following IS*5* insertion, consists of two parts of DNA with one part being from the adjacent genomic region that harbors a Crp-binding site upstream of the inserted IS*5* element, and the other from the inactive IS*5* transposase promoter. It is next interesting to know if this Crp site is important to P*_fsn_*’s functionality.

### The fusion promoter is activated by Crp bound to the upstream Crp-binding site *O_Crp_0*

**Figure 8.**
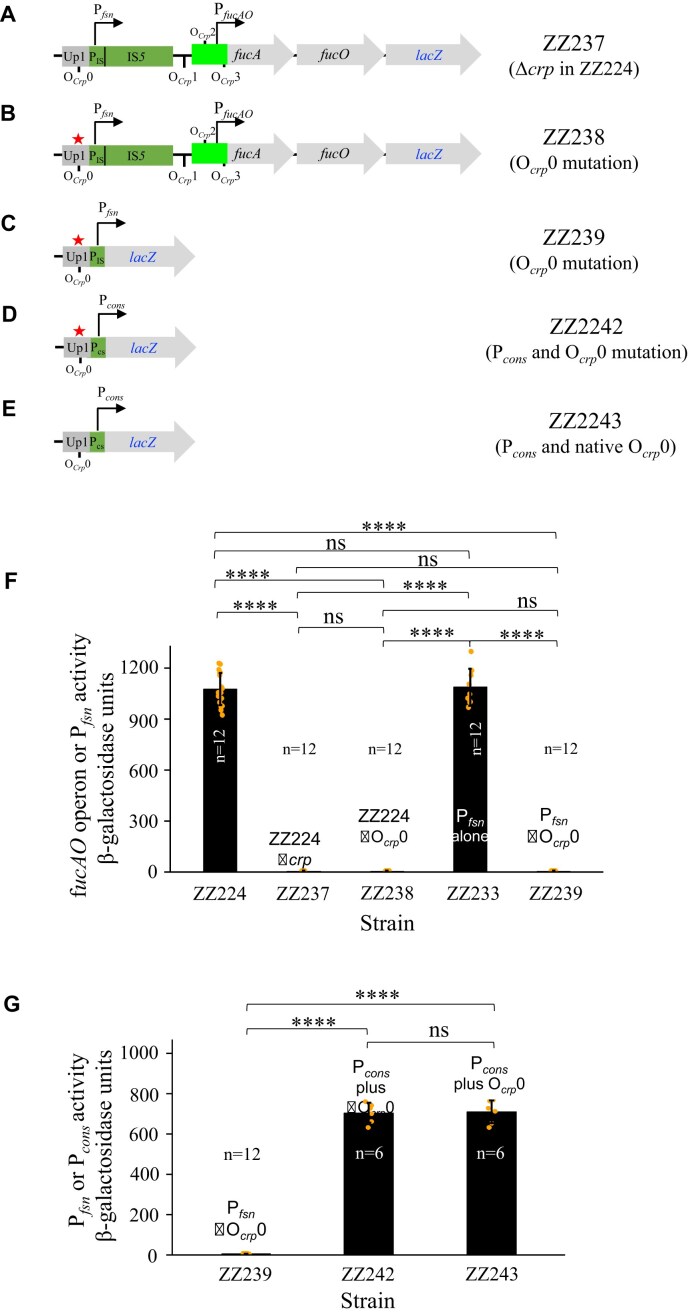
Global regulator Crp-cAMP and the upstream Crp-binding site O*_Crp_*0 are required for P*_fsn_* activity. (**A**) Diagram showing strain ZZ237 that is identical to strain ZZ224 but in a Δ*crp* background. (**B**) Diagram showing the alteration of O*_Crp_*0 upstream of P*_fsn_* in ZZ238, in which P*_fsn_*, IS*5*, P*_fucAO_* and *fucAO* are present. (**C**) Diagram showing the alteration of O*_Crp_*0 upstream of P*_fsn_* in ZZ239, in which P*_fsn_* alone drives *lacZ*. (**D**) Diagram showing the alteration of O*_Crp_*0 upstream of P_IS_ and the substitution of consensus sequences for the −35/−10 hexamers within P_IS_ in ZZ242, in which P*_cons_* (P_IS_ with consensus −35/−10 hexamers) alone (with no O*_Crp_*0) drives *lacZ*. P*_cs_* denotes the modified P_IS_ (P*_cons_*) carrying the consensus −35/−10 motifs. (**E**) Diagram showing the substitution of consensus sequences for the −35/−10 hexamers within P_IS_ in ZZ243, in which P*_cons_* together with the upstream O*_Crp_*0 drives *lacZ*. (**F**) Effects of Δ*crp* and O*_Crp_*0 mutations on *fucAO* operon transcription or the P*_fsn_* promoter activity under non-inducing conditions. (**G**) β-galactosidase assays, revealing that the modified P_IS_ (P*_cons_*) with consensus −35/−10 motifs not only exhibits a significantly increased promoter activity but also becomes independent of Crp. P*_cons_* denotes the modified P_IS_, in which the proposed −35/−10 motifs are replaced with the consensus −35/−10 motifs. Data are plotted as the mean ± SD (one-way ANOVA with Tukey Kramer's post-hoc test).

IS*5* insertion has long been thought to cause constitutive expression of the *fucAO* operon, independently of any known regulators. Crp and FucR are two primary positive regulators for the *fuc* regulon, and it is therefore worthwhile to determine if these regulatory proteins are involved in IS*5*-mediated *fucAO* operon activation. To consider a possible effect of Crp, we first transferred the operon *lacZ* reporter cassette together with the upstream IS*5* element to strain Δ*crp* Glp^+^ (a *crp* deletion strain able to grow on glycerol) [17]. When this Δ*crp* strain (ZZ237) (Fig. 8A) was cultured with glycerol, the operon was essentially without expression (Fig. 8F), indicating that Crp-cAMP is crucial for IS*5* activation of the *fucAO* operon.

We next determined which Crp-binding site(s) is/are involved in *fucAO* operon activation by IS*5*. There are four Crp-binding sites (O*_Crp_*0, O*_Crp_*1, O*_Crp_*2, and O*_Crp_*3) in the *fucPIK*/*fucAO* intergenic region [4] (Figs 1 and 8A). Among these binding sites, only O*_Crp_*0 is situated upstream of the inserted IS*5* element while O*_Crp_*1, O*_Crp_*2, and O*_Crp_*3 are located downstream of IS*5* in the *fucAO* regulatory region. These three downstream binding sites, O*_Crp_*1, O*_Crp_*2, and O*_Crp_*3, appear not to be important for IS*5*-mediated *fucAO* expression under non-inducing conditions since the lack of these sites does not affect operon expression (see Fig. 6B and F).

To determine if O*_Crp_*0 is involved in operon activation, it was altered by changing its two binding motifs (see Section 2.6) in strain ZZ224 (harboring IS*5*, P*_fucAO_* and *fucAO*) (Fig. 8B). The resultant strain ZZ238 is the same as strain ZZ224 except that O*_Crp_*0 was altered. As shown in Fig. 8F, like the Δ*crp* strain, mutation of O*_Crp_*0 abolished operon transcription, indicating that O*_Crp_*0 is essential for *fucAO* operon expression upon IS*5* insertion.

Based on the above findings, it is highly likely that the binding of Crp-cAMP to O*_Crp_*0 directly activates P*_fsn_*. To further confirm this possibility, the same O*_Crp_*0 mutation as for strain ZZ238 was made in strain ZZ233, yielding strain ZZ239 in which P*_fsn_* alone drives the *lacZ* reporter gene at the *fuc* locus (Fig. 8C). As expected, the lack of O*_Crp_*0 essentially eliminated P*_fsn_*’s activity (Fig. 8F), indicating that the direct binding of the Crp-cAMP complex to the upstream O*_Crp_*0 site is required for P*_fsn_* activation. In other words, *fucAO* operon expression, mediated by IS*5* insertion, is not constitutive and instead depends on Crp-cAMP binding to the newly generated fusion promoter, thus recruiting RNA polymerase (RNAP) to initiate operon transcription.

To further confirm that the *ins5A* promoter P_IS_ is a weak σ70 promoter and its activity relies on Crp, the −10/−35 motifs of P_IS_ within P*_fsn_* were changed to the consensus sequences. With such changes, the resultant promoter P*_cons_* (denoting the modified P_IS_ carrying the consensus −35/−10 hexamers) would conceivably not be dependent on any activators. To confirm this assumption, strains ZZ242 and ZZ243 were made, in which P*_cons_* plus ΔO*_Crp_*0 and P*_cons_* plus the native O*_Crp_*0 drive *lacZ*, respectively (Fig. 8D and E). P*_cons_* promoter activities were quantified with ZZ242 and ZZ243 under the non-inducing conditions in comparison to the activity of P*_fsn_* with the mutated O*_Crp_*0 (ZZ239) which has essentially no activity. As shown in Fig. 8G, P*_cons_* alone with no O*_Crp_*0 in strain ZZ242 exhibited 750-units of LacZ activity, an over 200-fold increase from P*_fsn_* without O*_Crp_*0 in ZZ239, indicating that the presence of the consensus −35/−10 motifs within the *ins5A* promoter P_IS_ dramatically increases the promoter activity. In the presence of the native upstream O*_Crp_*0, P*_cons_* had a similar activity to the same promoter lacking the O*_Crp_*0, indicating that the presence of O*_Crp_*0 does not favor the strength of P*_cons_*. Interestingly, P*_cons_* with or without O*_Crp_*0 (about 750 units) showed a 35% lower promoter activity than the functional P*_fsn_* (P_IS_ plus O*_Crp_*0) (about 1200 units; see Fig. 6D). This could be attributed to certain specific DNA sequences present in P*_cons_*, limiting the promoter activity. Nevertheless, these results support the notion that P_IS_ is a poor σ70 promoter, but it is regulatable by host transcriptional factors such as Crp-cAMP in this study.

### The fusion promoter activity is independent of FucR

**Figure 9.**
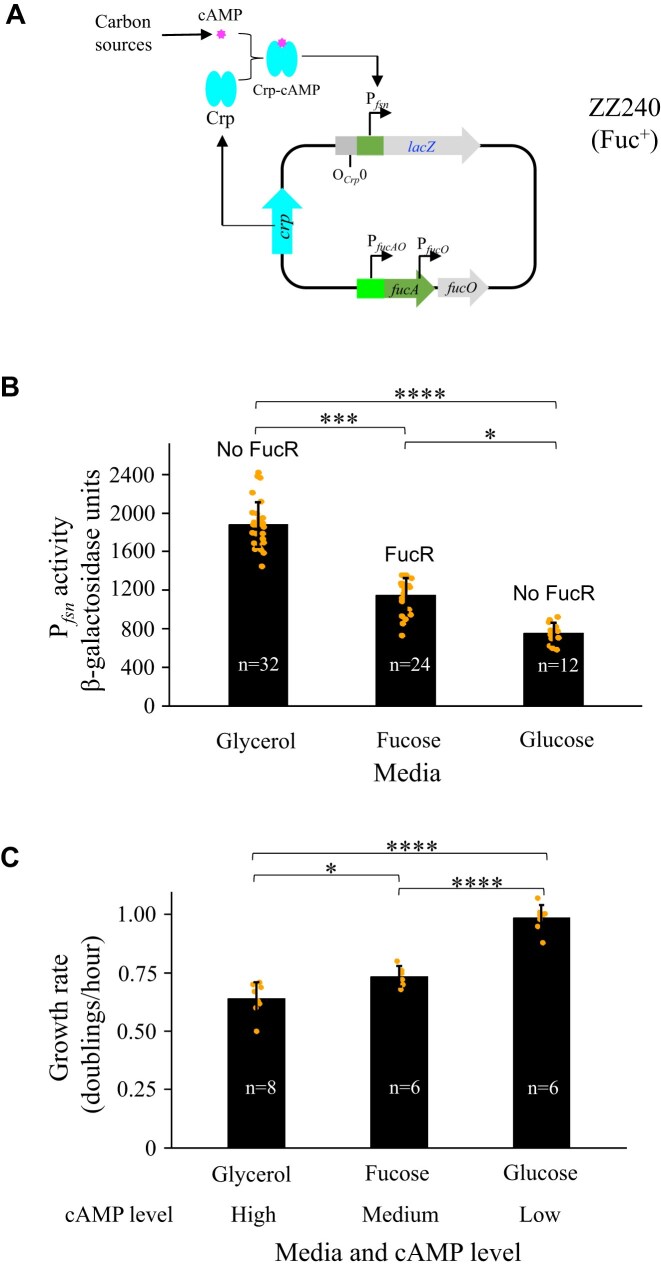
P*_fsn_* activity is independent of FucR. (**A**) A schematic diagram showing that P*_fsn_* alone drives *lacZ* at the *lac* locus in strain ZZ240 (Fuc^+^) while the native *fuc* regulon is intact. P*_fsn_* is solely regulated by Crp-cAMP. The cAMP levels depend on growth rates mediated by carbon sources with higher levels of cAMP present within slower growing cells [33]. (**B**) P*_fsn_* promoter activities in cells of strain ZZ240 cultured with glycerol, fucose or glucose in M63 minimal media. Active FucR is available in fucose-grown cells but not in glycerol or glucose-grown cells. (**C**) Growth rates of strain ZZ240 in minimal M63 media with glycerol, fucose or glucose as the carbon source. Data are plotted as the mean ± SD (one-way ANOVA with Tukey Kramer's post-hoc test).

The previous section demonstrated the Crp-dependence of the fusion promoter P*_fsn_* formed upon IS*5* insertion. We next examined whether FucR, the *fuc* regulon-specific activator, has an effect on P*_fsn_*. To do this, a new reporter strain ZZ240 (Fuc^+^) was made, in which P*_fsn_* alone drives *lacZ* at the *lac* locus, leaving the native *fuc* regulon intact (Fig. 9A). The promoter activities were measured when this new reporter strain was cultured in minimal media with glycerol (which does not activate FucR) and fucose (which does activate FucR), respectively (Fig. 9B). In the absence of FucR (glycerol media), P*_fsn_* exhibited 1880 units of promoter activity. In the presence of FucR (fucose media), the same promoter did not show additional activity, indicating that the activated FucR does not exert a stimulatory effect on P*_fsn_*. This result agrees with the fact that no known FucR binding site is present within or upstream of P*_fsn_* (Figs 6A and 9A).

The lower-than-expected promoter activity (i.e. 1248 units) observed with fucose grown cells compared with glycerol grown cells (1880 units) may be due to the presence of a lower amount cAMP due to catabolite repression. To indirectly confirm this assumption, the same cells were cultured with glucose minimal medium, under which cAMP is known to be synthesized at a low level [33] in addition to the absence of activated FucR. As expected, the glucose grown cells have an even lower P*_fsn_* activity (716 units) than fucose-grown cells (Fig. 9B). The cytoplasmic cAMP levels have been reported to be inversely correlated with growth rates in response to carbon sources, with lower cAMP amounts present in faster-growing cells [34]. As shown in Fig. 9C, strain ZZ240 had a 76-min doubling time when cultured in fucose medium, which is lower than when cultured in glycerol medium (91 min) but greater than when cultured in the glucose medium (58 minutes) (Fig. 9C). Based on these results, we conclude: (i) P*_fsn_* is not positively regulated by FucR and instead is exclusively activated by Crp-cAMP; and (ii) The P*_fsn_* strength depends on the cytoplasmic levels of cAMP, which varies (as expected) with the carbon sources.

At the *lac* locus, P*_fsn_* appeared to have nearly twice the amount of activity (1900 units; Fig. 9B) in glycerol-grown cells compared to the *fuc* locus (about 1050 units; Fig. 5F). This observation is consistent with our previous observation that the *fuc* locus is embedded within a transcriptionally repressed region on the chromosome [4].

### FucR can further elevate expression of the *fucAO* operon that is activated upon IS*5* insertion

**Figure 10.**
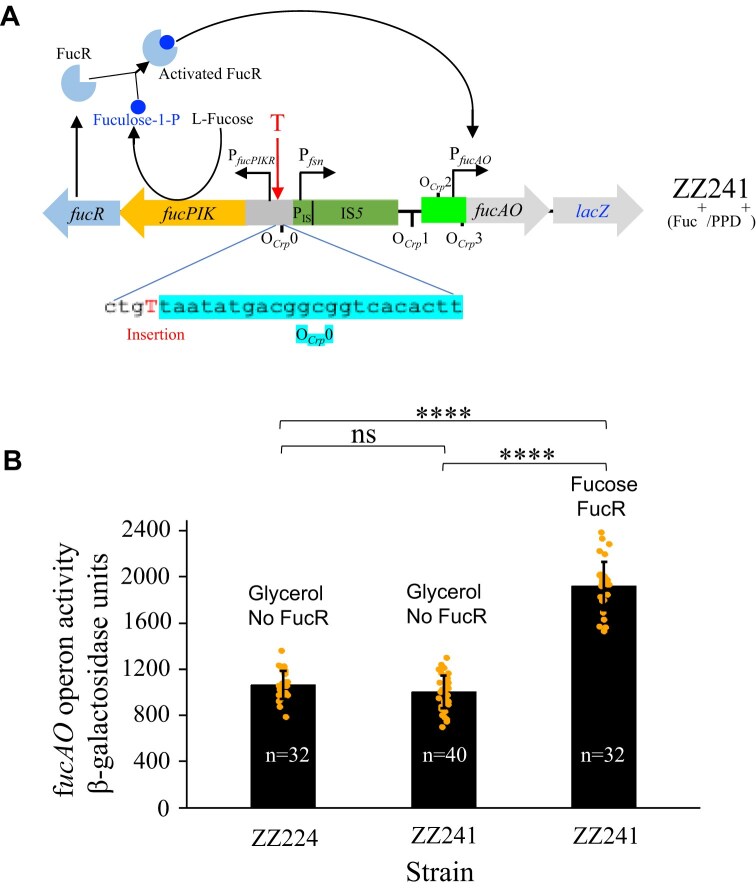
Active FucR can enhance *fucAO* operon transcription in IS*5* insertional PPD^+^ cells. (**A**) Schematic illustration showing a single nucleotide “T” insertion immediately downstream of O*_Crp_*0 within P*_fucPIKR_* of ZZ224 (PPD^+^), leading to a PPD^+^/Fuc^+^ phenotype (ZZ241). Activated FucR is available with the cells of ZZ241 cultured with fucose although it is absent when cultured with glycerol. (**B**) The *fucAO* operon activities with strain ZZ241 cultured under non-inducing or inducing conditions. Data are plotted as the mean ± SD (one-way ANOVA with Tukey Kramer's post hoc test).

It is known that with IS*5* insertion, the *fuc* regulon becomes non-inducible by fucose because the insertion inactivates *fucPIK* (and most likely *fucR* as well), leading to an extremely low level of FucR and a lack of L-fuculose-1-P, the inducer of FucR. Therefore, there is essentially no activated FucR present in IS*5* insertional PPD^+^ cells under any growth conditions ± fucose. To see whether FucR has a positive effect on *fucAO* operon expression, it should be necessary to render the activated FucR available. To do this, a Fuc^+^ PPD^+^ double mutant (ZZ241) was generated by prolonged incubation of IS*5* insertional PPD^+^ cells (Fuc^−^ PPD^+^) on minimal M9 + fucose agar plates. This double growth positive mutant carries an insertion of a single nucleotide, T, at position −146, upstream of the *fucP* translational start site (immediately downstream of O*_Crp_*0), and no other mutations were found within the *fuc* regulon (Fig. 10A). When cultured with fucose, this Fuc^+^/PPD^+^ strain was expected to produce activated FucR regulatory proteins since the *fucPIKUR* operon is expressed, and the inducer, fuculose-1-P, should be synthesized.

When this double mutant was cultured with glycerol (FucR is not activated), an operon activity comparable to that of the original PPD^+^ cells (that is ZZ224) was detected (Fig. 10B), indicating that the point mutation, causing a Fuc^+^ phenotype, does not affect IS*5* mediated *fucAO* operon expression. However, when this same double mutant was cultured with fucose (activated FucR should be present), a near two-fold operon activity increase was observed in comparison to the same strain cultured with glycerol (where no activated FucR should be present), indicating that FucR, when present in its activated form, can further elevate expression of the *fucAO* operon post-IS*5* insertion. The greater operon activity (that is, about 2140 units of β-galactosidase activity) observed for Fuc^+^/PPD^+^ cells with activated FucR present appears to be attributed to two separated promoters: the fusion promoter P*_fsn_* and the native operon promoter P*_fucAO_*. It is worth noting that the combined P*_fsn_*/P*_fucAO_* promoter activity (that is, 2140 units of LacZ activity) in Fuc^+^/PPD^+^ cells is slightly lower than the additive activity (that is 2300 units) of P*_fsn_* alone (1250 units) (Fig. 6) and P*_fucAO_* alone (1050 units) [4]. This might be due to the binding of Crp-cAMP and FucR to their operators upstream of P*_fucAO_*, which is inhibitory to P*_fsn_*. Based on these observations, we conclude: (i) The fucose-non-inducibility of IS*5* insertional PPD^+^ cells is due to the absence of activated FucR; (ii) Activated FucR, when present, is capable of further enhancing expression of the *fucAO* operon upon IS*5* insertion although its binding partially blocks P*_fsn_* initiated transcription; and (iii) when IS*5* insertion and the activated FucR are simultaneously present (e.g. Fuc^+^/PPD^+^ cells grown in fucose media), P*_fsn_* and P*_fucAO_* function nearly independently and account for transcription of the *fucAO* operon nearly equally.

## Discussion

We have demonstrated that activation of the otherwise silent *fucAO* operon in *E. coli* by IS*5* insertion is attributable to the formation of a functional fusion promoter P*_fsn_*, consisting of the beginning region of IS*5* as a basic self-inactive promoter and a short genomic region with a Crp-binding site O*_Crp_*0 immediately upstream of the inserted element as the activating site. Once bound to O*_Crp_*0, Crp-cAMP activates P*_fsn_*, which in turn is responsible for the transcription of the downstream *fucAO* operon. IS*5* has been reported to activate or elevate gene/operon expression by various means, including de-repression, native promoter activation, and DNA loop disruption. In this report, we have discovered a novel IS*5* activation mechanism by creating a fusion promoter involving a host chromosomal region. This newly formed promoter dictates the entire *fucAO* operon transcription, and its activity exclusively depends on Crp-cAMP but not on FucR. Under the inducing conditions, functional FucR can further elevate *fucAO* operon expression by activating the native operon promoter. In this case, the fusion promoter and the native promoter drive operon transcription independently and nearly equally. These results not only fill in the gap in our yet-to-be known knowledge concerning IS*5* activation of the *fucAO* operon but also provide further insight into the complex mechanisms underlying the gene/operon regulation upon transpositions of transposable elements.

Forming fusion promoters is a commonly used mechanism for other IS elements to positively affect expression of target genes/operons, especially for those conferring multidrug resistances in bacteria. IS*1* insertion activates a β-lactamase gene (leading to ampicillin resistance) by forming a σ70 fusion promoter with the “−35″ element from the right end of IS*1* (terminal inverted repeat on the right side, IRR) and the “−10″ element from the native gene promoter [35]. IS*2* insertion upstream of the *acrAB* operon results in the formation of a fusion promoter, with the −35 hexamer derived from the IRR of IS*2* and the −10 hexamer from the native promoter, leading to increased fluoroquinolone resistance [36, 37]. Formation of similar σ70 fusion promoters (with the −35 motif from the IRR and the −10 motif from the downstream flanking region), activating or promoting the transcription of nearby muti-drug resistance genes, have been documented for numerous other IS elements as well, including IS*6*, IS*21*, IS*30*, IS*256*, IS*982*, and IS*Aba825* [38–43]. In addition, Tf1, a retrotransposon, has been reported to increase transcription of several stress response genes in yeast by providing an enhancer-like element [44]. Surprisedly, little has been known as to any fusion promoters formed using a terminal IS region with an upstream genomic region. The functional fusion promoter formed upon IS*5* insertion within the *fucPIK*/*fucAO* intergenic region is unique in that it carries an upstream genomic region as an activating site and a 5′ end region of the inserted IS*5* element as a basic promoter. The activation of this otherwise inactive promoter within IS*5* is reliant on the adjacent upstream genomic region. To our knowledge, similar activation mechanisms have not been reported for this or any other bacterial transposable elements.

The TSS and the −10 motif of the fusion promoter identified in this study correspond to those previously proposed for the IS*5* transposase gene (*ins5A*) [30]. Based on the sequence of P*_fsn_* and some common criteria for a typical σ70 promoter, we proposed a −35 motif within IS*5*’s IRL as part of the *ins5A* promoter, which is located 17 bps upstream of the −10 motif (Fig. 7C). Both the −10 and −35 motifs within the *insA* promoter are dissimilar from the consensus promoter sequences, suggesting its low-level activity. Using a β-galactosidase reporter, our previous study indicated that the *ins5A* promoter alone has essentially no activity [16]. All these observations agree with each other, pointing to the fact that the *ins5A* gene can barely be transcribed from its native promoter. Therefore, prior to *ins5A* transcription, the responsive promoter needs to be activated by a genomic element, which is most likely derived from the upstream chromosomal region. This is the case for the IS*5*-mediated fusion promoter characterized in this study: an activating element (O*_Crp_*0) situated in the upstream genomic region of the basic promoter carrying a −35 motif, a −10 motif and a TSS from the 5′ end of IS*5*. As expected, the resultant promoter drives transcription not only of *ins5A* but also of the downstream *fucAO* operon. An additional observation worthy of attention is the disparity between the transcriptional start site (TSS) located at nucleotide “A” identified in our investigation, and the two TSSs (“C” and “T”) reported in a previous study [30].

Our study employed the 5′RACE method in contrast to the primer extension method utilized in the prior research to discover the TSS. Given that our TSS is located merely one nucleotide downstream of the proposed “C” TSS, it is plausible that inaccuracy may have occurred during the initial identification of this TSS using the primer extension method. Alternatively, the TSS identified in the current study might represent a new one specifically for the newly generated fusion promoter.

Fusion promoters generated upon insertion of transposable elements are usually constitutively active, and their activities do not rely on any known transcriptional activators from the host [36, 45, 46]. IS*5* insertion upstream of the *fucAO* operon has long been assumed to cause constitutive operon expression. However, we demonstrated that this hybrid promoter alone exhibits no activity in the absence of the *crp gene*, and its activation depends on Crp-cAMP bound to the upstream Crp-binding site, O*_crp_*0. Like other sugar metabolic operon promoters, this promoter is subject to catabolite repression (that is, lower activity observed for glucose-grown cells than glycerol or fucose-grown cells). These results indicate that *fucAO* operon expression upon IS*5* insertion is not “constitutive” but instead is positively regulated by Crp-cAMP, the global activator for >180 genes/operons involved in carbon metabolism [47–50].

In summary, the functionality for the fusion promoter identified in this study depends on the following molecular properties: (i) the presence a Crp-binding site O*_Crp_*0 in the adjacent genomic region upstream of the inserted IS*5* element; (ii) the presence of an otherwise inactive promoter at the right-side end of IS*5*; (iii) a proper distance (preferentially 17 to 18 bps) between the −35 motif and the −10 motif; and (iv) a proper distance (i.e. 14 bps) between O*_Crp_*0 and the −35 motif. To satisfy these requirements, IS*5* must insert at a proper genomic site (with a single direction), buried in a SIDD structure. Therefore, the results reported in this study can well explain why the inserted IS*5* elements are always found at the same position and in the same orientation (with the transposase gene *ins5A* transcribed in the same direction as *fucAO*) in each identified PPD^+^ mutant [11].

Crp activated promoters are categorized into three classes. In Class I promoters, Crp binds upstream of the promoter at sites centered to positions −61.5, 71.5, 82.5, or 92.5 with respect to the TSS. For these promoters, Crp activates transcription by directly interacting with the C-terminal domain of RNAP α subunit (α-CTD) [51, 52]. For Class II promoters, Crp binds to the upstream site centered at position −41.5, overlapping the −35 element [52, 53]. For these promoters, Crp activation of transcription relies on at least two contacts with RNAP: one with the α-CTD, facilitating RNAP binding to the promoter, and the other with the *N*-terminal domain of the α subunit (α-NTD), facilitating subsequent promoter opening. For Class III Crp-dependent promoters, Crp binds to two or more sites upstream of the promoter. These promoters are usually dependent on a second regulator that helps Crp recruit RNAP [52, 54, 55]. As shown in Fig. 7C, the Crp-binding site O*_Crp_*0 within P*_fsn_* is centered at position −61.5 upstream of the TSS, and O*_Crp_*0 is the only binding site upstream of the fusion promoter. The further upstream region is dispensable for promoter activity, and there are no known binding sites for other regulators. Based on these feathers, we conclude that the fusion promoter P*_fsn_* formed upon IS*5* insertion into the *fucPIKUR*/*fucAO* intergenic region is a typical Crp-dependent Class I promoter.

Our results show that the fusion promoter alone exhibits 15% greater activity than the complete IS*5* element together with the downstream *fucAO* operon in the PPD^+^ cells. This could be due to the presence of the other three Crp-binding sites downstream of IS*5*. Once bound to these downstream sites, Crp-cAMP might serve as a weak roadblock protein that somewhat slows down transcription. However, this blockage impact appears to be limited since the native operon promoter remains inactive in the presence of the IS*5* insertion. Alternatively, a possible DNA or mRNA structure, when present downstream of P*_fsn_*, may negatively affect operon expression as well.

Besides Crp, FucR is the only other activator known for the *fuc* regulon. We have clarified that the fusion promoter itself is FucR independent as there is no FucR binding site present upstream of this promoter (all four known binding sites are located downstream of the inserted IS*5* element). However, we provided evidence that FucR, when present in its active form, is capable of further enhancing *fucAO* operon transcription relative to that driven by the fusion promoter, and this additional transcription is attributed to the native operon promoter that is activated by FucR. This result reveals that within IS*5* insertional PPD^+^ cells, the *fucAO* operon can be inducible (as is true for wild-type cells) when the active FucR is available. One such condition is observed when PPD^+^/Fuc^+^ double mutants are grown under inducing conditions. Double growth-positive mutants can readily occur by prolonged incubation of PPD^+^ cells with fucose as the sole carbon source. Taken together, these findings provide an example of how bacterial cells can evolve the genetic capability to adapt to changing environments.

With IS*5* insertion upstream of the *fucAO* operon, the PPD^+^ cells not only carry an additional copy of IS*5* in their chromosomes but most likely also express the IS*5* transposase gene at a higher level, which might lead to an increased frequency of IS*5* transposition. In addition, the transposition would possibly be subject to Crp-cAMP regulation since the P*_fsn_* activity is heavily reliant on this global regulator. However, the consequence (if any) caused by *ins5A* overexpression may be compromised by the following observations. First, the IS*5* transposase mainly exerts a *cis* effect, being biased to transpose the element that synthesizes the enzyme [56]. Second, this IS5 element has been shown to precisely excise from the *fucPIK*/*fucAO* intergenic region under a starvation condition although it is at a low frequency [11]. Based on these observations, the increased *ins5A* expression due to IS5 insertion upstream of P*_fucAO_* might not cause a severe consequence to the PPD^+^ cell. Apparently, more studies are needed to examine the outcomes of elevated amounts of transposases within the PPD^+^ cells as to their impacts on overall transposition and their implications in bacterial physiology.

While our study has successfully identified the molecular mechanism responsible for IS*5*-mediated activation of the *fucAO* operon, there remains a gap in our understanding of the initial insertion event and the later excision event involving IS*5*. Therefore, a potential avenue for future research is to investigate the environmental conditions that promote IS*5* transposition into or IS*5* excision out of this specific location. Meanwhile, future studies are needed to examine if Crp and FucR, two primary activators of the *fuc* regulon, play regulatory roles in IS*5* insertion or excision. These studies will not only provide a more comprehensive understanding of the regulation of the *fucAO* operon but should also shed light on the broader mechanisms that govern transposon movement within bacterial genomes.

## Supplementary Material

gkaf172_Supplemental_Files

## Data Availability

The data underlying this article will be shared on reasonable request to the corresponding author.
