# Supplementary material for: Transcriptional mechanism by which IS5 activates the fucAO operon in Escherichia coli
Source: Nucleic Acids Res. 2025 Mar 11;53(5):gkaf172. doi: 10.1093/nar/gkaf172 (PMC11894529; doi:10.1093/nar/gkaf172)
Supplement: gkaf172_Supplemental_Files [file gkaf172_supplemental_files.zip › Supplementary Table 1_Rev_final_0208.pdf]

Supplementary Table 1. Strains used in this study

Strains	Genotype or description	Reference
BW25113	Wild type, *lacI*^{q} *rrnB*$_{T14}$ Δ*lacZ*$_{WJ}$ Δ*hsdR*514 Δ*araBAD*$_{AH33}$ Δ*rhaBAD*$_{LD78}$	(30)
ZZ200	Δ*lacI*, Δ*lacZ* and Δ*lacY* in BW25113	(4)
PPD$^+$	IS5 insertion upstream of P_{fucAO}; aerobic growth on PPD	(13)
PPD$^+$Δ*lacZ*	Δ*lacI*, Δ*lacZ* and Δ*lacY* in PPD$^+$	This study
ZZ204	Carrying a *lacZ* gene plus its own RBS immediately downstream of *fucO* in Δ*lacZ*; Cm^r	(4)
ZZ224	Carrying a *lacZ* gene plus its own RBS immediately downstream of *fucO* in PPD$^+$Δ*lacZ*; Cm^r	This study
ZZ225	Substitution of a *km*^{r} gene for IS5 in ZZ224	This study
ZZ226	Chromosomal insertion of a *km*^{r} gene in ZZ204 at the same site as for IS5 in ZZ224	This study
ZZ227	Substitution of *km*^{r} for the 5' part of IS5 except IB in ZZ224	This study
ZZ228	Δ*km*^{r} in ZZ227	This study
ZZ229	Deleting the first 68 bps from 5' end of IS5 (ΔP_{IS}) in ZZ224	This study
ZZ230	Deleting the first 100-bp region upstream of IS5 (ΔUp1) only in ZZ224	This study
ZZ231	Deleting the second 100-bp region upstream of IS5 (ΔUp2) only in ZZ224	This study
ZZ232	Up2 and Up1 directly driving *lacZ* (deleted for IS*5*, P_{fucAO} and *fucAO* in ZZ224)	This study
ZZ233	P_{fsn} alone driving *lacZ* at the *fuc* locus	This study
ZZ234	P_{fsn} with the entire IS5 driving *lacZ* at the *fuc* locus	This study
ZZ235	Adding a *rrnB* terminator downstream of P_{fsn} in ZZ224	This study
ZZ236	Adding a *rrnB* terminator between P_{fucAO} and *fucA* in ZZ224	This study
ZZ237	Δ*crp*(Glp$^+$) in ZZ224	This study
ZZ238	O_{Crp}0 mutation upstream of P_{fsn} in ZZ224	This study
ZZ239	O_{Crp}0 mutation upstream of P_{fsn} in ZZ233	This study
ZZ240	P_{fsn} alone driving *lacZ* at the *lac* locus, Fuc$^+$	This study
ZZ242	P_{cons} with the mutated O_{Crp}0 driving *lacZ* at the *fuc* locus in ZZ233	This study
ZZ243	P_{cons} with the native O_{Crp}0 driving *lacZ* at the *fuc* locus in ZZ233	This study
ZZ241	PPD$^+$/Fuc$^+$ with a "T" insertion downstream O_{Crp}0 in ZZ224	This study
