# Supplementary material for: Transcriptional mechanism by which IS5 activates the fucAO operon in Escherichia coli
Source: Nucleic Acids Res. 2025 Mar 11;53(5):gkaf172. doi: 10.1093/nar/gkaf172 (PMC11894529; doi:10.1093/nar/gkaf172)
Supplement: gkaf172_Supplemental_Files [file gkaf172_supplemental_files.zip › Supplementary Table 2_Rev_final_0208.pdf]

Supplementary Table 2. Oligonucleotides used in this study

Name	Sequence	Use
IS5-km-P1	cattttcaaataaaaaacgctctgtaatatgacggcggtcacacttattagtgtgtagg ctggagctgcttc	Replacing IS5 with *km*^{r} in strain ZZ224 to make ZZ225or inserting *km*^{r} at the IS5 insertion site in strain ZZ204 to make ZZ226
IS5-km-P2	catgaaatttcgattattaaagtgatggtagtcacataaagtcaccttctagattccggg gatccgtcgacctg	Replacing IS5 with *km*^{r} in strain ZZ224 to make ZZ225or inserting *km*^{r} at the IS5 insertion site in strain ZZ204 to make ZZ226
PfucAO-ver-F	ttcgggcttcatatcagtgaatatttgcctg	Verification of *km*^{r} substitution for IS5 and *km*^{r} insertion
fucA-ver-R1	atcccatcctgataacgtacactgac	Verification of *km*^{r} substitution for IS5 and *km*^{r} insertion
IB-km-P1	tcaaataaaaaacgctctgtaatatgacggcggtcacacttattagctagtgtgtagg ctggagctgcttc	Replacing 5' region of IS5 except IB with *km*^{r} in strain ZZ224 to make ZZ227
IB-km-P2	taaggcgttatccccagtttttagtgagatctctcccactgacgtatcattattccgggg atccgtcgacctg	Replacing 5' region of IS5 except IB with *km*^{r} in strain ZZ224 to make ZZ227
PIS-km-P1	ttcaaataaaaaacgctctgtaatatgacggcggtcacacttattagctagtgtgtagg ctggagctgcttc	Replacing the 1st 68-bp 5' end region of IS5 with *km*^{r} in strain ZZ224 to make ZZ229
PIS -km-P2	cggcgcttactgctgaattcactgtcggcgaaggtaagttgatgactcatattccggg gatccgtcgacctg	Replacing the 1st 68-bp 5' end region of IS5 with *km*^{r} in strain ZZ224 to make ZZ229
Up1-km-P1	agcacgtaatcggttatgttttcaaaagtgcaaaaccagcgagttcgggctgtgtag gctggagctgcttc	Replacing the 1st 100-bp region upstream of IS5 with *km*^{r} in strain ZZ224 to make ZZ230
Up1-km-P2	gctccagatgacaaacatgatctcatatcagggacttgttcgcaccttccattccggg gatccgtcgacctg	Replacing the 1st 100-bp region upstream of IS5 with *km*^{r} in strain ZZ224 to make ZZ230
Up2-km-P1	tggctaccgcccaaagaaaaaacagtgagcacagcagcgcgaatggaatatgtgt aggctggagctgcttc	Replacing the 2nd 100-bp region upstream of IS5 with *km*^{r} in strain ZZ224 to make ZZ231
Up2-km-P2	gcccgaactcgctggttttgcacttttgaaaacataaccgattacgtgctattccgggg atccgtcgacctg	Replacing the 2nd 100-bp region upstream of IS5 with *km*^{r} in strain ZZ224 to make ZZ231
Up-lacZ-F	ttcaaataaaaaacgctctgtaatatgacggcggtcacacttattagctagcacagga aacagctatgaccatgattacg	Substitution of "*lacZ*:*cat*" for IS5/P$_{fucAO}$/*fucAO* in ZZ224 to make ZZ232
Up-cat-R	gcatatcgacggcaatttacagcaattgcggttggtacggtaacggcgagcttacgc cccgccctgccactcatcgcag	Substitution of "*lacZ*:*cat*" for IS5/P$_{fucAO}$/*fucAO* in ZZ224 to make ZZ232
Pfsn-lacZ-F	cctgatatgagatcatgtttgtcatctggagccatagaacagggttcatccacaggaa acagctatgaccatgattacg	Substitution of "*lacZ*:*cat*" for IS5/P$_{fucAO}$/*fucAO* except P$_{IS}$ in ZZ224 to make ZZ233
Pfsn-cat-R	ttgcatatcgacggcaatttacagcaattgcggttggtacggtaacggcgagcttac gccccgccctgccactcatcgc	Substitution of "*lacZ*:*cat*" for IS5/P$_{fucAO}$/*fucAO* except P$_{IS}$ in ZZ224 to make ZZ233
IS5-lacZ-F	gaaatgactgagtcagccgagaagaatttccccgcttattcgcaccttcccacagga aacagctatgaccatgattacg	Substitution of "*lacZ*:*cat*" for P$_{fucAO}$/*fucAO* in ZZ224 to make ZZ234
IS5-cat-R	tgcatatcgacggcaatttacagcaattgcggttggtacggtaacggcgagcttacg ccccgccctgccactcatcgca	Substitution of "*lacZ*:*cat*" for P$_{fucAO}$/*fucAO* in ZZ224 to make ZZ234

Pfsn-T1-F	cctgatatgagatcatgtttgtcatctggagccatagaacagggttcatctgtgtaggc tggagctgcttc	Insertion of a *rrnB* terminator downstream of P$_{fsn}$ in ZZ224 to make ZZ235
Pfsn-T1-R	cggcgcttactgctgaattcactgtcggcgaaggtaagttgatgactcataaaggttc atcgcgctcgagacgca	Insertion of a *rrnB* terminator downstream of P$_{fsn}$ in ZZ224 to make ZZ235
IS5-T1-F	gaaatgactgagtcagccgagaagaatttccccgcttattcgcaccttcctgtgtagg ctggagctgcttc	Insertion of a *rrnB* terminator downstream of IS5 in ZZ224 to make ZZ236
IS5-T1-R	tttctgaaacgggcatgaaatttcgattattaaagtgatggtagtcacataaaggttcat cgcgctcgagacgca	Insertion of a *rrnB* terminator downstream of IS5 in ZZ224 to make ZZ236
GST-fucA-R	tttccaggcaagtgtcaataatctgacgag	*fucA* specific oligo used for 5’RACE
GST-IS5-R	tgttttgccatggcagaatctgctccatg	IS5 specific oligo used for 5’RACE
GST-lacZ-R	tcttcgctattacgccagctggc	*lacZ* specific oligo used for 5’RACE
Up1-km-F	agcacgtaatcggttatgttttcaaaagtgcaaaaccagcgagttcgggctgtgtag gctggagctgcttc	Amplification of “*km*^{r}:*rrnB*T”, and fusion of “*km*^{r}:*rrnB*T” and “Up1-O$_{Crp}$0”
Km-T-R	ggcaaatattcactgatatgaaaggttcatcgcgctcgagacgc	Amplification of “*km*^{r}:*rrnB*T”
Ocrp0-mut-F	gcgtctcgagcgcgatgaacctttcatatcagtgaatatttgcc	Amplification of Up1 region with mutated O$_{Crp}$0
Ocrp0-mut-R	caaacatgatctcatatcagggacttgttcgcaccttccctagctaataagctagctaa taagaacatccgccgaggtattacagag	Amplification of Up1 region with mutated O$_{Crp}$0, fusion of “*km*^{r}:*rrnB*T” and “Up1-O$_{Crp}$0”
Km-Pfsn-F	gcatttacgttgacaccatcgaatggcgcaaaacctttcgcggtatgtgtaggctgga gctgcttc	Amplification of “*km*^{r}:*rrnB*T”, and fusion of “*km*^{r}:*rrnB*T” and “P$_{fsn}$-*lacZ*`”
Km-Pfsn-R	caggcaaatattcactgatatgaagaaaggttcatcgcgctcgagacgc	Amplification of “*km*^{r}:*rrnB*T”
Pfsn-F	gcgtctcgagcgcgatgaacctttcttcatatcagtgaatatttgcctg	Amplification of P$_{fsn}$-*lacZ*` from ZZ233
lacZ’-R	tctggtgccggaaaccaggcaaagcg	Amplification of P$_{fsn}$-*lacZ*` from ZZ233, and fusion of “*km*$^{r}$:*rrnB*T” and “P$_{fsn}$-*lacZ*`”
LacZ-R3	gatgtgctgcaaggcgattaag	Verification of P$_{fsn}$ driving *lacZ* at the lac locus
km-Up-P1	agcacgtaatcggttatgttttcaaaagtgcaaaaccagcgagttcgggctgtgtag gctggagctgcttc	Replacing the “Up1:P$_{IS}$” region with a *km*^{r} gene in strain ZZ233
P$_{IS}$-km-P2	ccagtcacgacgttgtaaaacgacggccagtgaatccgtaatcatggtcatattccg gggatccgtcgacctg	Replacing the “Up1:P$_{IS}$” region with a *km*^{r} gene in strain ZZ233
km-T -F	agcacgtaatcggttatgttttcaaaagtgcaaaaccagcgagttcgggctgtgtag gctggagctgcttc	Amplification of “*km*^{r}:*rrnB*T”
Km-T-R	gaaaatgaatccatgagttcatttcagacagaaaggttcatcgcgctcgagacgc	Amplification of “*km*^{r}:*rrnB*T”
Up-F	gcgtctcgagcgcgatgaacctttctgtctgaaatgaactcatggattcattttc	Amplification of “P$_{cons}$`”
Pcons-R	ctccagatgacaaacattatatcatatcagggacttgttgtcaacttccctagc	Amplification of “P$_{cons}$`”
Pcons-F	gctagggaagttgacaacaagtccctgatatgatataatgtttgtcatctggag	Amplification of “`P$_{cons}$-*lacZ*`”
lacZ-R100	tgcgggcctcttcgctattacgccagctg	Amplification of “`P$_{cons}$-*lacZ*`”
